# Causal relationship between bulimia nervosa and microstructural white matter: evidence from Mendelian randomization

**DOI:** 10.1007/s40519-025-01754-z

**Published:** 2025-05-19

**Authors:** Yiling Wang, Xinghao Wang, Jiani Wang, Weihua Li, Qian Chen, Zhanjiang Li, Lirong Tang, Marcin Grzegorzek, Wenjuan Liu, Zhenchang Wang, Peng Zhang

**Affiliations:** 1 Department of Radiology, Beijing Friendship Hospital, Capital Medical University, Beijing, 100050 China; 2 Institute for Medical Informatics, University of Luebeck, 23562 Luebeck, Germany; 3 Beijing Anding Hospital Capital Medical University, Beijing, 100088 China; 4 The National Clinical Research Center for Mental Disorders & Beijing Key Laboratory of Mental Disorders, Beijing Anding Hospital, Capital Medical University, Beijing, 100088 China; 5 Department of Radiology, Aerospace Center Hospital, Beijing, 100049 China; 6 Peking University Aerospace School of Clinical Medicine, Beijing, 100049 China

**Keywords:** Bulimia nervosa, White matter, Diffusion magnetic resonance imaging, Mendelian randomization, Causal effect

## Abstract

**Purpose:**

Observational studies suggest white matter (WM) microstructural anomalies are linked to bulimia nervosa (BN), but a direct causal relationship remains unestablished. This study aimed to investigate the causal impact of BN on WM microstructure.

**Methods:**

We analyzed genome-wide association study (GWAS) summary data from 2442 individuals to identify genetically predicted BN. Diffusion MRI were obtained from the UK Biobank. After assessing instrumental variable validity, we conducted Mendelian randomization (MR) using inverse variance weighting (IVW) as the primary method, followed by pleiotropy and heterogeneity tests.

**Results:**

The MR analysis from BN to brain imaging-derived phenotypes showed that BN had significant causal effects on a union set of nine tracts (including a total of 18 image-derived phenotypes) (IVW, *P* < 0.05): brainstem tracts (pontine crossing tract, bilateral medial lemniscus, left superior cerebellar peduncle, and middle cerebellar peduncle), sensory-related tracts (right retrolenticular part of the internal capsule and left inferior longitudinal fasciculus), and emotion-related tracts (left anterior corona radiata and right cingulum hippocampus).

**Conclusion:**

This study revealed that BN has a causal effect on WM microstructure, which extends the reports of association to causation for WM and BN. These causal effects may explain the deficits in feeding, taste, vision, and emotion regulation that are often observed in patients with BN.

*Level of evidence III* well-designed cohort analytic study.

**Supplementary Information:**

The online version contains supplementary material available at 10.1007/s40519-025-01754-z.

## Introduction

Bulimia nervosa (BN) is a psychiatric disorder characterized by recurrent binge eating episodes that are accompanied by compensatory behaviors to control body weight [1]. Individuals with BN may develop several medical and psychiatric comorbidities that can increase the risk of premature mortality and suicide [2, 3]. With existing treatments, only 30–40% of BN patients achieve therapeutic remission, which may be attributed to the unclear neuropathological mechanisms [1].

Numerous structural and functional magnetic resonance imaging studies have uncovered widespread functional and grey matter structural abnormalities in the cortical and subcortical regions of BN patients [4–7]. For example, localized neuronal abnormalities in the inferior parietal lobule and insula and abnormal gray matter volume in the medial orbitofrontal cortex have also been reported in patients with BN [5, 6]. Wang et al. [4] revealed that the BN-related alterations in subcortical striatal functional connectivity with three key regions: the dorsolateral prefrontal cortex implicated in self-regulation, the thalamus involved in taste reward, as well as the visual occipital and sensorimotor regions supporting body image. Another study [7] also identified significant functional network reorganization of the nucleus accumbens, an important subcortical structure, with the frontal cortex in BN patients. Of note, brain white matter (WM) axons connect cortical and subcortical structures and thus play a crucial role in cortical–subcortical information integration [8]. Recently, there has been an increase in studies focused on WM alterations in BN; however, findings have been inconsistent: Wagner et al. [9] found no significant differences in either total or regional WM volume in BN patients compared with healthy controls. While other studies reported that BN patients had impaired WM integrity in temporoparietal regions associated with vision [10] and regions associated with taste and reward processing [11]. He et al. [12] revealed the abnormal WM microstructural properties in BN patients utilizing the tract-based spatial statistics (TBSS) analysis. In addition, using TBSS and automated fiber quantification, we previously found significant reorganization of WM microstructure in BN patients, yet observed no alterations in WM volume or properties of the major WM tracts [13]. While growing evidence from observational studies links BN to WM structural changes, these findings cannot establish causality due to confounding factors and reverse causation. Mendelian randomization (MR) can bridge this gap by leveraging genetic variants to infer causal relationships between BN and WM alterations.

MR analysis is an epidemiological study approach that uses the results of a genome-wide association study (GWAS) for directional causal inference [14]. MR utilizes the fixed nature of genes and Mendelian’s first and second laws of inheritance, which state that during meiotic gamete formation, parent’s alleles are randomly assigned to the offspring, and the relationship between genes and outcomes is unaffected by common confounding factors, such as postnatal environment, socioeconomic factors, and behavioral habits. MR is the process of employing a single nucleotide polymorphism (SNP) as a variable instrument and using randomly allocated genes in nature to infer the influence of phenotype on illness [15]. Randomization refers to the strong resemblance between MR and randomized controlled trials and their ability to demonstrate a causal relationship between modifiable exposures and illness outcomes. MR allows the identification of causal and directional relationships with outcomes by using genetic variations linked to changeable qualities or exposures. Recent studies have demonstrated the value of applying MR to examine the causal effects of psychiatric or degenerative illnesses on brain abnormalities [16, 17].

Thus, to address the gap in the knowledge regarding the causal relationship between BN and WM, we aimed to investigate the causal effect of BN on microstructural WM using diffusion magnetic resonance imaging (dMRI) and MR. Our findings may be crucial for understanding the causal mechanism underlying the disruption of WM microstructure in BN patients.

## Materials and methods

**Fig. 1:**
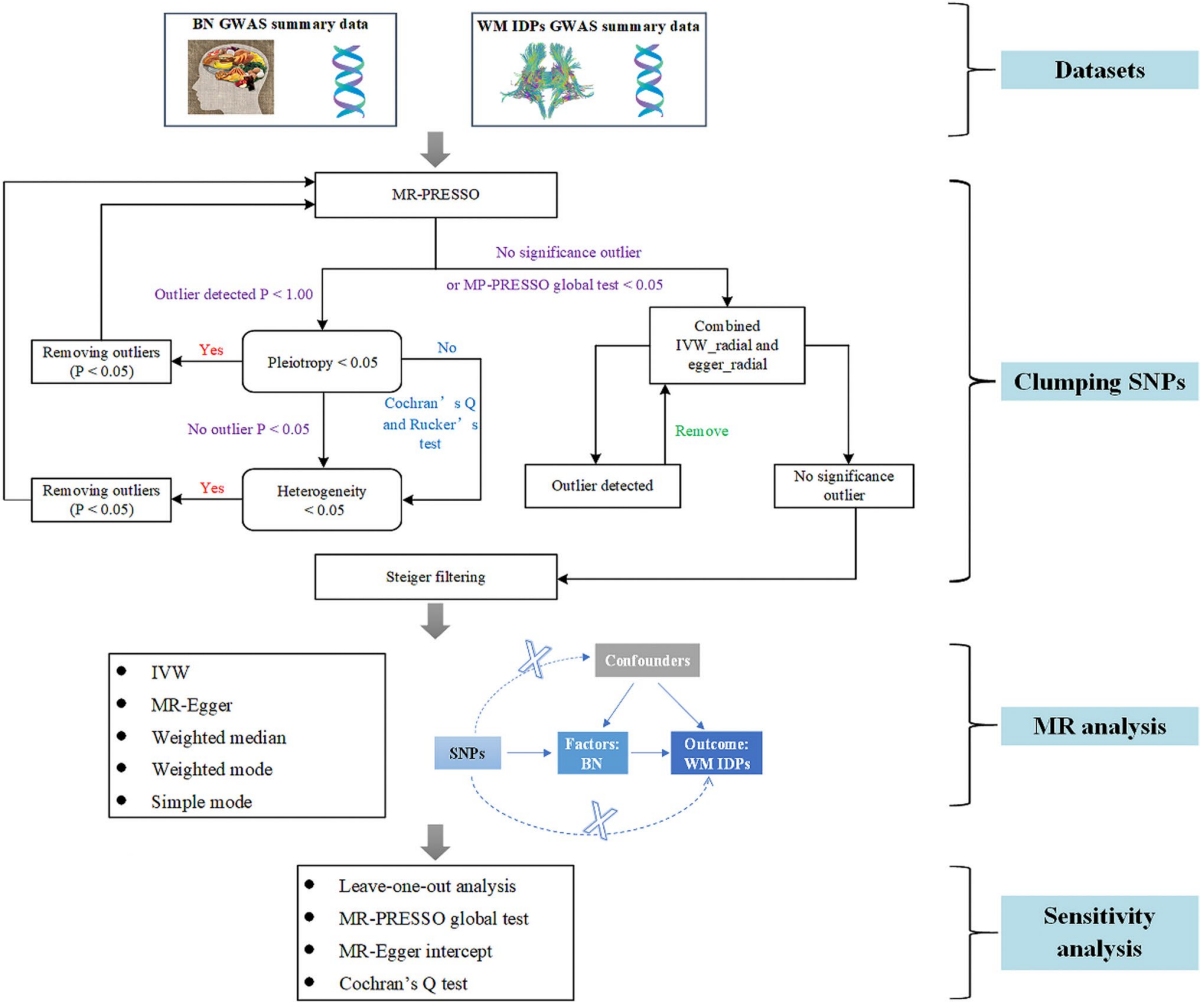
Overall workflow diagram. *BN* bulimia nervosa, *GWAS* genome-wide association study, *WM* white matter, *IDPs* imaging-derived phenotypes, *MR-PRESSO* Mendelian randomization Pleiotropy Residual Sum and Outlier, *IVW* inverse variance weighting, *SNPs* single nucleotide polymorphisms

We applied two-sample MR to explore the effect of BN on WM microstructure. We followed the strobe-MR statement [18] for the MR analysis process. In addition, our procedures were conducted in compliance with the process definition of MR research. Figure 1 shows the study design and process.

### GWAS of BN

The GWAS of BN was based on a genetic study on eating disorders [19]. The participants were obtained from adult volunteers in the National Health and Medical Research Council Australian Twin Registry. The data were collected from two groups of people who responded to a poll distributed by post between 1988 and 1992. The dataset included genetic and eating disorder conditions as well as sociodemographic information, which included age, marital status, educational background, employment rate, significant lifetime jobs, and religious affiliations. The study initially analyzed 7262007 SNPs (only *R*^2^ quality control testing), filtered out SNPs with a minor allele frequency <2%, and selected the final SNPs that represented BN according to symptom characteristics. The GWAS data from BN patients fully accounted for the influence of a long-term cohort and growth on diet. Age and characteristics associated with the cohort (marital status, educational background, workforce participation, major lifetime occupation, and religious denomination) were entered as covariates in the GWAS analysis. Finally, the GWAS data of BN patients comprised 2442 individuals and 11846513 SNPs, which represented the genetic characteristics of the BN population (Supplementary material 1).

### GWAS of WM microstructure

The representative values of brain WM microstructure were obtained from dMRI data in the UK Biobank [14]. The data were acquired in 50 diffusion-encoding directions for the two diffusion-weighted shells (and all 100 directions were distinct). A typical (“monopolar”) Stejskal-Tanner pulse sequence was used for the dMRI data acquisition, which has a shorter echo duration (echo time = 92 ms) than a twice-refocused (“bipolar”) sequence and thus a higher signal-to-noise ratio. The stronger eddy current distortions caused by this sequence were mitigated using tools within the image processing pipeline. The dMRI data were first adjusted for eddy currents, head motion, and outlier slices using the Eddy tool (http://fsl.fmrib.ox.ac.uk/fsl/fslwiki/EDDY). Then, gradient distortion correction was applied to generate four-dimensional output data. The generated data were then analyzed using two complementary methods: TBSS, which is based on tract-skeleton processing, and probabilistic tractography, which is based on a more detailed within-voxel tract structure model. Both analysis streams yielded a variety of dMRI-derived metrics for specific tract regions. These metrics were: (A) diffusion tensor modeling measurements and (B) microstructural model fitting measures. The dMRI subfolders contained the outputs of both models. The *b* = 1000 shell (50 directions) was input into the diffusion tensor imaging (DTI) fitting tool DTIFIT, which produced DTI outputs, such as fractional anisotropy (FA), mean diffusivity (MD), and axial diffusion (AD). The FA reflects unrestricted diffusion of water reflecting cellular damage, and the MD stands for the speed of diffusion and changes in membrane permeability [20, 21]. Decreased FA and increased MD reflect compromised white matter integrity, potentially caused by changes in axonal diameter, fiber density, tissue organization, myelination levels, and elevated extracellular free water [22, 23]. The AD specifically measures water diffusion parallel to axons, and increased AD has been associated with axonal degeneration [24, 25]. In addition, the dMRI data underwent neurite orientation dispersion and density imaging (NODDI) modeling using the AMICO software (https://github.com/daducci/AMICO) to provide significant voxel-wise microstructural indices, such as the orientation dispersion index (OD), isotropic or free water volume fraction (ISOVF), and intracellular volume fraction (ICVF). The ISOVF represents the isotropic volume fraction (i.e., unrestricted diffusion) and usually calculates the extent of cerebrospinal fluid contamination, the ICVF represents the intracellular volume fraction and is highly correlated with axon density and integrity, and the OD describes the spatial dispersion of neurite structures and estimates the orientation consistency of neurites [26, 27]. In general, a higher OD value suggests the crossing of neurites [28]. A higher ISOVF value is usually expected in neuroinflammatory states [29]. And a lower ICVF value indicates loss or damage of neurites [30]. Subsequently, they examined the relationship between the genes and neurological imaging parameters and interpolate missing values (at a threshold of −log10 (*P*) > 11) for each of the 3144 GWASs conducted. Finally, we selected 675 manifestations pertaining to WM microstructure in this study, which are detailed in Supplementary Table S1.

### MR analysis and quality control

To investigate the causal effect of BN on WM microstructure, we used two-sample MR. To determine whether BN impacts WM microstructure, we first selected closely related SNPs from the BN GWAS results (*P* threshold: 5 × 10^−6^). The MR Pleiotropy Residual Sum and Outlier (MR-PRESSO) global test was applied to identify the outliers (i.e., SNPs with *P* < 0.05). If horizontal pleiotropy was detected, the outliers were removed, and MR-PRESSO was run again. Subsequently, using the SNPs that survived pleiotropy correction, we evaluated the between-SNP heterogeneity by applying the inverse variance weighting (IVW) and MR-Egger approaches. To confirm the presence of heterogeneity, we used Cochran’s Q and Rucker’s Q (for the MR-Egger approach). During this stage, if there was substantial heterogeneity and horizontal pleiotropy was absent, we eliminated SNPs with *P* < 1.00 in the MR-PRESSO analysis [31] and reran MR-PRESSO. If the number of SNPs exceeded five, the radial-MR approach was applied. If none of the detected SNPs were significant in the MR-PRESSO analysis, or if the global test *P*-value was less than 0.05 when the MR-PRESSO analysis was conducted [32], the radial IVW and Egger methods were then used to further identify and remove outliers with *P* < 0.05. The IVW and Egger approaches were reapplied once the outliers were eliminated or until no outliers could be found. Subsequently, we used Steiger filtering to exclude SNPs that accounted for a larger percentage of the variation in the result than that in the exposure when both pleiotropy and heterogeneity were deficient [33]. After obtaining the selected representative SNPs, we conducted analyses using a variety of MR methods (mainly IVW) [34]. In addition, the MR results were tested for heterogeneity among the SNPs included in each analysis using Cochran’s Q-test. The detection of horizontal pleiotropy relied on the intercept of MR Egger. A leave-one-out analysis was used to identify the influential SNPs.

The statistical analysis and data visualization, including MR, were conducted using the TwoSampleMR, RadialMR, MendelianRandomization, GenomicSEM, GSMR, and MRPRESSO packages in the R software (version 4.3.2). The platform used for opening and analysis was RStudio (https://posit.co/products/open-source/rstudio/), which is an integrated programming environment for R and Python. Following the above steps, there were different instrumental variables for each leak and outcome, which were not considered repeated measurements or observations. A *P* < 0.05 on both sides was deemed statistically significant.

## Results

### Overview of the study

**Fig. 2:**
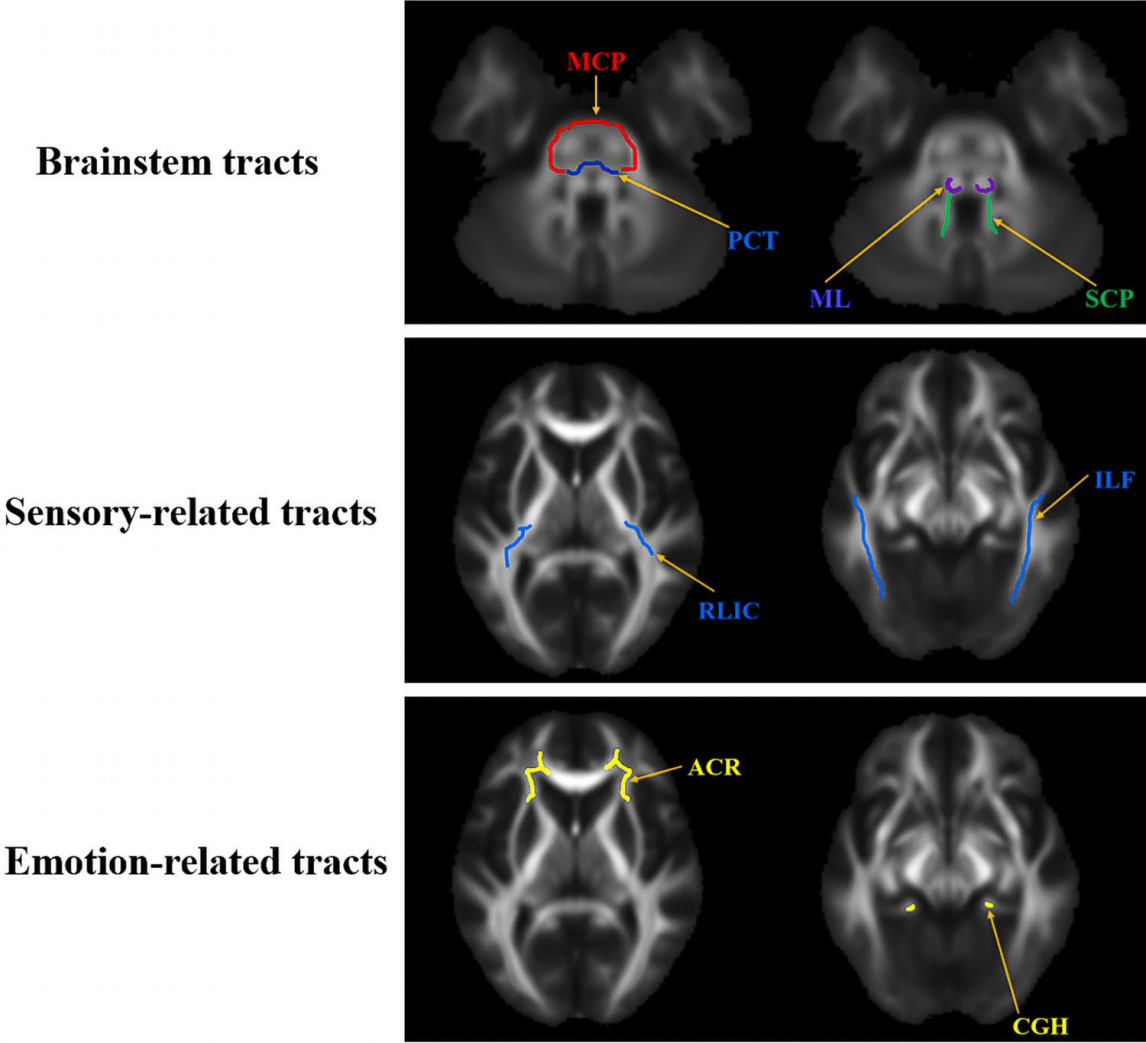
Anatomical schematic of white matter fibers causally associated with BN. *MCP* middle cerebellar peduncle, *PCT* pontine crossing tract, *ML* medial lemniscus, *SCP* superior cerebellar peduncle, *RLIC* retrolenticular part of the internal capsule, *ILF* inferior longitudinal fasciculus, *ACR* anterior corona radiate, *CGH* cingulum hippocampus

After filtering the SNPs, a total of 34 SNPs of BN were selected (Table 1). As shown in Table 2 and Supplementary Table S2, the primary IVW analysis demonstrated a significant causal effect of BN on 18 WM imaging-derived phenotypes (IDPs) (*P* < 0.05). The SNP information and statistical power of each MR analysis are listed in Supplementary Table S3. Detailed MR analysis results of these IDPs are provided in Supplementary material 2 (Figure A series). The anatomical locations of the WM fibers that were causally associated with BN are illustrated in Fig. 2, and they are classified into different categories according to their anatomy and function as shown in Table 3.

**Table 1:** Threshold-filtered SNP list from bulimia nervosa GWAS data

SNPs	beta.exposure	pval.exposure	eaf.exposure
rs985795	0.094	2.22E−06	0.054
rs17021865	0.193	4.38E−06	0.011
rs4665166	0.330	9.78E−08	0.007
rs35345871	0.314	3.00E−06	0.004
rs1445130	0.056	1.08E−07	0.136
rs7570172	1.736	3.75E−09	0.005
rs10498122	0.175	2.57E−06	0.049
rs10461209	2.806	5.25E−11	0.001
rs35355482	0.241	4.27E−06	0.061
rs62290592	0.226	2.73E−06	0.016
rs299362	−0.062	2.52E−06	0.887
rs2881621	1.605	1.65E−06	0.002
rs1556640	−0.075	2.33E−06	0.879
rs28631020	0.08	3.45E−06	0.077
rs17141485	0.229	8.32E−08	0.013
rs16911431	0.933	1.67E−08	0.004
rs62528694	0.524	9.14E−07	0.005
rs55948608	0.427	2.31E−06	0.003
rs12246837	0.578	4.69E−06	0.01
rs7903789	0.74	4.94E−06	0.002
rs11215626	0.482	9.74E−07	0.002
rs12222192	1.933	4.16E−07	NA
rs61910395	0.233	2.97E−06	0.014
rs7957608	0.282	3.21E−06	0.01
rs9564662	0.243	1.27E−07	0.014
rs2589311	0.8	4.92E−10	0.011
rs35240311	0.884	4.99E−09	0.002
rs45487297	0.822	1.39E−06	0.006
rs12590626	0.657	1.95E−07	0.002
rs8040855	0.035	3.32E−06	0.64
rs55644649	0.398	3.95E−07	0.02
rs12986207	0.044	3.90E−06	0.184
rs8104126	1.475	9.90E−07	0.003
rs6135470	0.527	3.81E−06	0.005

*SNP* single nucleotide polymorphism, *GWAS* genome-wide association study

**Table 2:** Results of the two-sample Mendelian randomization (MR) analysis using bulimia nervosa as the exposure

Exposure	Outcome	SNPs	IVW-derived *P* value	IVW-derived β value (95% CI)	se	Cochran’s Q test *P* value	MR-Egger intercept test *P* value
BN	The MD of pontine crossing tract^#^	29	1.38 × 10^–4^	0.051 (0.025∼0.078)	0.014	0.876	0.121
	The MD of right medial lemniscus^#^	31	1.32 × 10^–2^	0.034 (0.007~0.061)	0.014	0.851	0.076
	The AD of pontine crossing tract^#^	29	8.41 × 10^–5^	0.051 (0.026~0.077)	0.013	0.885	0.057
	The AD of right medial lemniscus^#^	31	1.18 × 10^–3^	0.046 (0.018~0.073)	0.014	0.779	0.467
	The AD of left medial lemniscus^#^	32	1.64 × 10^–2^	0.033 (0.006~0.060)	0.014	0.924	0.639
	The AD of left superior cerebellar peduncle^#^	32	4.89 × 10^–2^	0.022 (0.0001~0.044)	0.011	0.836	0.826
	The AD of left anterior corona radiata^#^	32	3.35 × 10^–2^	−0.027 (−0.052~−0.002)	0.013	0.794	0.760
	The AD of right medial lemniscus*	29	1.37 × 10^–2^	0.038 (0.008~0.068)	0.015	0.834	0.428
	The ISOVF of pontine crossing tract^#^	31	4.23 × 10^–5^	0.056 (0.029~0.083)	0.014	0.832	0.724
	The ISOVF of right medial lemniscus^#^	32	9.63 × 10^–3^	0.033 (0.008~0.059)	0.013	0.871	0.566
	The ISOVF of left medial lemniscus^#^	34	2.98 × 10^–2^	0.027 (0.003~0.051)	0.012	0.939	0.364
	The ISOVF of left superior cerebellar peduncle^#^	32	4.76 × 10^–2^	0.025 (0.0002~0.049)	0.013	0.709	0.252
	The ISOVF of right retrolenticular part of internal capsule^#^	33	7.40 × 10^–3^	−0.037 (−0.063~−0.010)	0.014	0.628	0.416
	The OD of right medial lemniscus^#^	29	3.91 × 10^–2^	−0.030 (−0.059~−0.002)	0.015	0.337	0.136
	The OD of left medial lemniscus^#^	30	3.13 × 10^–2^	−0.030 (−0.057~−0.003)	0.014	0.584	0.162
	The OD of right cingulum hippocampus^#^	33	7.57 × 10^–3^	0.036 (0.010~0.062)	0.014	0.993	0.881
	The OD of left inferior longitudinal fasciculus*	31	5.71 × 10^–3^	0.037 (0.011~0.062)	0.013	0.994	0.932
	The OD of middle cerebellar peduncle*	31	2.46 × 10^–2^	0.031(0.004~0.058)	0.014	0.853	0.084

*IDPs* brain imaging-derived phenotypes, *MD* mean diffusivity, *AD* axial diffusion, *OD* orientation dispersion index, *ISOVF* isotropic or free water volume fraction, *IVW* inverse-variance weighted

^#^The outcome white matter imaging-derived phenotypes were derived from the tract-based spatial statistics (TBSS) analysis

*The outcome white matter imaging-derived phenotypes were derived from the probabilistic tractography analysis

**Table 3:** The classification for the causal effects of bulimia nervosa on white matter

Classification	Tract	Altered dMRI parameter
Brainstem tract	Pontine crossing tract	MD, AD, ISOVF
	Right medial lemniscus	MD, AD, ISOVF, OD
	Left medial lemniscus	AD, ISOVF, OD
	Left superior cerebellar peduncle	AD, ISOVF
	Middle cerebellar peduncle	OD
Sensory-related tract	Right retrolenticular part of internal capsule	ISOVF
	Left inferior longitudinal fasciculus	OD
Emotion-related tract	Left anterior corona radiata	AD
	Right cingulum hippocampus	OD

*dMRI* diffusion-weighted magnetic resonance imaging, *MD* mean diffusivity, *AD* axial diffusion, *OD* orientation dispersion index, *ISOVF* isotropic or free water volume fraction

### The causal effect of BN on WM IDPs from DTI data

The WM IDPs derived from DTI data were divided into IDPs that were analyzed using TBSS and those that were analyzed using probabilistic tractography. For the WM IDPs derived from TBSS, BN showed a significant causal effect on the increased MD of the pontine crossing tract and right medial lemniscus (IVW-β: 0.051 and 0.034; 95% confidence interval [CI]: 0.025 to 0.078 and 0.007 to 0.061, respectively; both *P* < 0.05). In addition, BN exhibited a causal effect on the increased AD in the pontine crossing tract, right medial lemniscus, left medial lemniscus and left superior cerebellar peduncle (IVW-β: 0.051, 0.046, 0.033 and 0.022; 95% CI: 0.026 to 0.077, 0.018 to 0.073, 0.006 to 0.060 and 0.0001 to 0.044, respectively; all *P* < 0.05), and a causal effect on the decreased AD in the left anterior corona radiata (IVW-β: −0.027; 95% CI: −0.052 to −0.002; and *P* < 0.05). For the IDPs derived from the probabilistic tractography analysis, BN had a causal effect on the increased AD in the right medial lemniscus (IVW-β: 0.038; 95% CI: 0.008 to 0.068; *P* < 0.05).

### The causal effect of BN on WM IDPs from NODDI data

The WM IDPs derived from NODDI data were also divided into IDPs that were analyzed using TBSS and those that were analyzed using probabilistic tractography. For the WM IDPs derived from TBSS, BN was causally associated with the increased OD of the right cingulum hippocampus (IVW-β: 0.036; 95% CI: 0.010 to 0.062; and *P* < 0.05) and causally related to the decreased OD of the right and left medial lemniscus (IVW-β: −0.030 and −0.030; 95% CI: −0.059 to −0.002 and −0.057 to −0.003; both *P* < 0.05). Furthermore, BN had a causal effect on the increased ISOVF of the pontine crossing tract, right medial lemniscus, left medial lemniscus, and left superior cerebellar peduncle (IVW-β: 0.056, 0.033, 0.027, and 0.025; 95% CI: 0.029 to 0.083, 0.008 to 0.059, 0.003 to 0.051, and 0.0002 to 0.049; all *P* < 0.05). Moreover, the ISOVF of the right retrolenticular part of the internal capsule was decreased due to BN (IVW-β: −0.037; 95% CI: −0.063 to −0.010; *P* < 0.05). For the IDPs derived from the probabilistic tractography analysis, the OD of the left inferior longitudinal fasciculus and middle cerebellar peduncle was increased by BN (IVW-β: 0.037 and 0.031; 95% CI: 0.011 to 0.062 and 0.004 to 0.058; both *P* < 0.05).

The quality control of the MR analysis results showed that all the above positive results passed the tests for heterogeneity and pleiotropy (as shown in Table 2). Details of the results of the pleiotropy and heterogeneity tests were presented in Supplementary material 2, where the funnel plots (Figure B series) represented the results of the pleiotropy test, the forest plots (Figure C series) showed the results of the heterogeneity test, and the leave-one-out analyses (Figure D series) provided additional supporting evidence for the pleiotropy test.

## Discussion

BN is a complex eating disorder, and although previous observational studies have shown associations between BN and WM microstructure, the causal relationship remains unclear. To address this knowledge gap, we investigated the causal relationship between BN and WM microstructural alterations using a two-sample MR-based analysis. Our primary findings were: (1) BN predominantly caused WM abnormalities in several brainstem fibers, which were reflected in various diffusion MRI parameters, suggesting that BN may contributes to disrupted information flow within the cerebrocerebellar circuit. (2) BN caused WM alterations in fibers involved in sensory processing, including taste and vision. (3) BN had a causal effect on the WM alterations of tracts relevant to emotion regulation.

In the present study, BN most often led to the WM abnormalities in the tracts in brainstem; alterations were observed for FA, MD, AD, OD, and ISOVF. Although these parameters are derived from different imaging methods and represent diverse or even contrasting significances [22, 35, 36], the implications represented by these parameters are not always absolute and may depend on the brain region, cellular basis, or sample studied [37]. These findings may suggest that BN has a causal effect on the microstructure of brainstem WM tracts. WM abnormalities of the brainstem tracts have also been consistently detected in patients with anorexia nervosa (AN) [37, 38]. The medial lemniscus, middle cerebellar peduncle, superior cerebellar peduncle, and pontine crossing tract are all important tracts that connect the cerebellum to the cerebral cortex and relay information between the two regions [38]. Recently, the cerebellum has been shown to be involved in feeding behaviors. Furthermore, it has been demonstrated to contribute to cognition, emotion, and behavior [39, 40], which are all crucial to the development and maintenance of BN. Notably, previous studies have reported volumetric alterations as well as disrupted functional connectivity in the cerebellum of patients with eating disorders [41–43]. Therefore, we speculate that BN interferes with the neuronal interconnections within the corticolimbic-cerebellar system, which is crucial for safeguarding cognition, emotion, and behavior. Thus, the multidimensional psychopathological difficulties encountered by BN patients, such as maladaptive feeding behaviors and emotional dysregulation, may be attributed to the impairment of the WM microstructure of brainstem fibers.

We also found that BN resulted in a decreased ISOVF of the right retrolenticular part of the internal capsule and an increased OD of the left inferior longitudinal fasciculus. Both tracts are associated with sensory processing. The internal capsule is the major conduit for brain information flow and contains ascending fibers from the thalamus to the cortex and descending fibers from the frontoparietal cortex to subcortical structures [44]. Decreased FA in the internal capsule has been detected in both patients with BN [11] and those with AN [38, 45, 46]. The thalamocortical projections of the internal capsule are primarily involved in the transmission of taste information [47], and these projections are disrupted in patients with central taste disorders [48]. Furthermore, deep brain stimulation targeting the internal capsule has been shown to alter taste perception [49]. Behavioral evidence [50] has revealed that patients with BN experience deficits in taste perception and reward. The ISOVF is a NODDI parameter that indexes the diffusivity of extracellular water molecules, and a decrease in ISOVF reflects an increase in myelination of the internal capsule [26]. Given the physiological functions of the internal capsule and the behavioral characteristics of BN, we suspect that the causal effect of BN on the decreased ISOVF of the internal capsule may contributes to patients’ impaired taste information transmission, which, in turn, results in taste disturbances. The inferior longitudinal fasciculus is a large association fiber connecting the anterior temporal lobe to the occipital cortex and is dedicated to visual processing [51]. Via et al. [52] reported that the FA of the inferior longitudinal fasciculus in patients with AN is lower than in healthy controls. Excessive preoccupation on body size and distorted body image perception is a central feature of BN [53]. A previous meta-analysis noted that both AN and BN patients overestimate their body size [54]. The OD, a parameter derived from NODDI data, indicates the dispersion of neurite fibers [26]. The increased OD value may suggest a disrupted fiber structure or demyelination [28]. BN results in an increase in the dispersion of neurite fibers in the left inferior longitudinal fasciculus, which may explain the enhanced local brain activity in the visual-related regions of BN patients observed previously [6, 55] and offer a structural basis for the inappropriate perception of body image in patients with BN.

We observed that BN caused an increase in the OD of the right cingulum hippocampus and a decrease in the AD of the left anterior corona radiata. Both tracts are involved in emotional processes, and WM alterations in these tracts have been documented in a wide range of psychiatric disorders characterized by emotional dysfunction [56–58]. Both abnormal AD and OD reflect disruption in WM integrity. Specifically, abnormal AD indicates axonal damage [35], whereas aberrant OD suggests abnormalities in synaptic plasticity [26]. Previous DTI studies on BN have also detected abnormalities in the two aforementioned tracts [11, 59]. The cingulum hippocampus is an important component of the Papez circuit, which is a well-recognized neural network that participates in a variety of limbic functions, especially emotion regulation [60]. The anterior corona radiata, which is part of the limbic-thalamo-cortical circuitry [61], is also involved in mood regulation. Mettler et al. [11] demonstrated that the anxiety trait of BN patients is correlated to the FA of the corona radiata. In addition, two previous studies in healthy samples revealed that anxiety traits are related to cortico-limbic WM integrity [62, 63]. Disturbed emotion regulation is common in BN patients with psychiatric disorders; indeed, a high proportion of BN patients have comorbid mood and anxiety disorders [64]. Taken together, the causal effect of BN on the microstructure of emotion-related fibers revealed in the present study may partially explain BN patients’ emotional disturbance.

Overall, by elucidating this causal relationship, our findings have the potential to identify specific WM tracts that are particularly susceptible to the effects of BN, thereby aiding in the exploration of therapeutic targets for neuromodulation in patients with BN. In addition, based on the physiological functions of these WM fibers (e.g., taste and emotion regulation), perhaps our findings can help in the development of targeted therapies aimed at alleviating some specific symptoms (e.g., binge eating, anxiety, etc.). However, the practice of these potential clinical applications still needs to be supported by a greater amount of research evidence.

Although we successfully identified a causal relationship between BN and WM abnormalities, the study has several limitations. First, the main statistical analysis method used was MR. The stability of MR results is influenced by various factors, such as genetic variable strength, heterogeneity, and pleiotropy. Although our results have been tested by relevant testing methods, we cannot blindly trust them. Several reports [65–67] advise caution and objectivity to be exercised when interpreting MR results. Second, for the GWAS of WM, dMRI parameters have lower genetic effectiveness than structural MRI parameters; therefore, diffusion data should be interpreted with caution. Third, even though we have used the largest currently available data set, the number of GWAS data on BN in this study may also lead to limitations in the results, namely that while the genetic validity of the GWAS was stated clearly, the sample size likely needs to be larger given the lack of positive results. Fourth, we must consider the various confounding factors or biases associated with BN, such as sex, other mental diseases, and the representativeness and independence of SNPs. Among them, for the results without potential multiple correction, we believe that the observation results are relatively independent, and there is no multiple correction in this paper, which is worth discussing in the future. Then, the results should be interpreted within a fair range [68]. The European demographic background is probably the most valuable consideration.

## Conclusion

We established using a two-sample MR analysis that BN has a causal effect on the WM microstructure of brainstem tracts, sensory-related tracts, and emotion-related tracts, and these causal effects may interpret patients’ disturbances in feeding, taste, vision, and emotion regulation. Our findings extend previous reports of associations between WM microstructure and BN by offering a causal dimension, which provide novel insight into the mechanisms underlying BN and promising targets for future precision treatments.

### Strength and limits

• First to study the investigate the causal impact of BN on WM microstructure.

• We found that BN has a causal effect on the WM microstructure of the brainstem tracts, sensory-related tracts, and emotion-related tracts.

• These findings extend previous reports of associations between WM microstructure and BN by offering a causal dimension, providing novel insight into the mechanisms underlying BN and promising targets for future precision treatments.

• The main analysis method used in this study is MR, the stability of its results is affected by many factors. Even though our results have been tested by relevant testing methods, we are cautious and objective in interpreting these results.

• Although we used the largest currently available dataset, the number of GWAS data on BN in this study may also have influenced the robustness of our findings, which warrants validation of our results with a larger sample in future studies.

### What is already known on this subject?

Although there is a growing body of studies linking BN to WM structural changes, they are essentially observational studies. The presence of various confounding factors and reverse causality in traditional observational studies presents challenges for examining the causal relationship between BN and WM alterations.

### What this study adds?

This study revealed that BN has a causal effect on WM microstructure, which extends the reports of association to causation for WM and BN. Our primary findings were: (1) BN predominantly caused WM abnormalities in several brainstem fibers, which were reflected in various diffusion MRI parameters, suggesting that BN contributes to disrupted information flow within the cerebrocerebellar circuit. (2) BN caused WM alterations in fibers involved in sensory processing, including taste and vision. (3) BN had a reliable causal effect on the WM alterations of tracts relevant to emotion regulation. These findings may provide novel insight into the mechanisms underlying BN and promising targets for future precision treatments.

## Supplementary Information

Below is the link to the electronic supplementary material.

Supplementary file1 (XLSX 56 KB)

Supplementary file2 (DOCX 310 KB)

Supplementary file3 (PDF 12422 KB)

## Data Availability

All data are publicly available. The GWAS resources come from IEU-OpenGWAS (https://gwas.mrcieu.ac.uk/）. For specific methods of brain imaging resources and data processing, please refer to the UK Biobank about the brain imaging offfcial website information: UK Biobank Brain Imaging (https://www.fmrib.ox.ac.uk/ukbiobank/index.html).
