# Supplementary material for: Causal relationship between bulimia nervosa and microstructural white matter: evidence from Mendelian randomization
Source: Eat Weight Disord. 2025 May 19;30(1):41. doi: 10.1007/s40519-025-01754-z (PMC12089160; doi:10.1007/s40519-025-01754-z)
Supplement: Supplementary file 2 — Supplementary file2 (DOCX 310 KB) [file 40519_2025_1754_MOESM2_ESM.docx]

**GWAS description of BN**


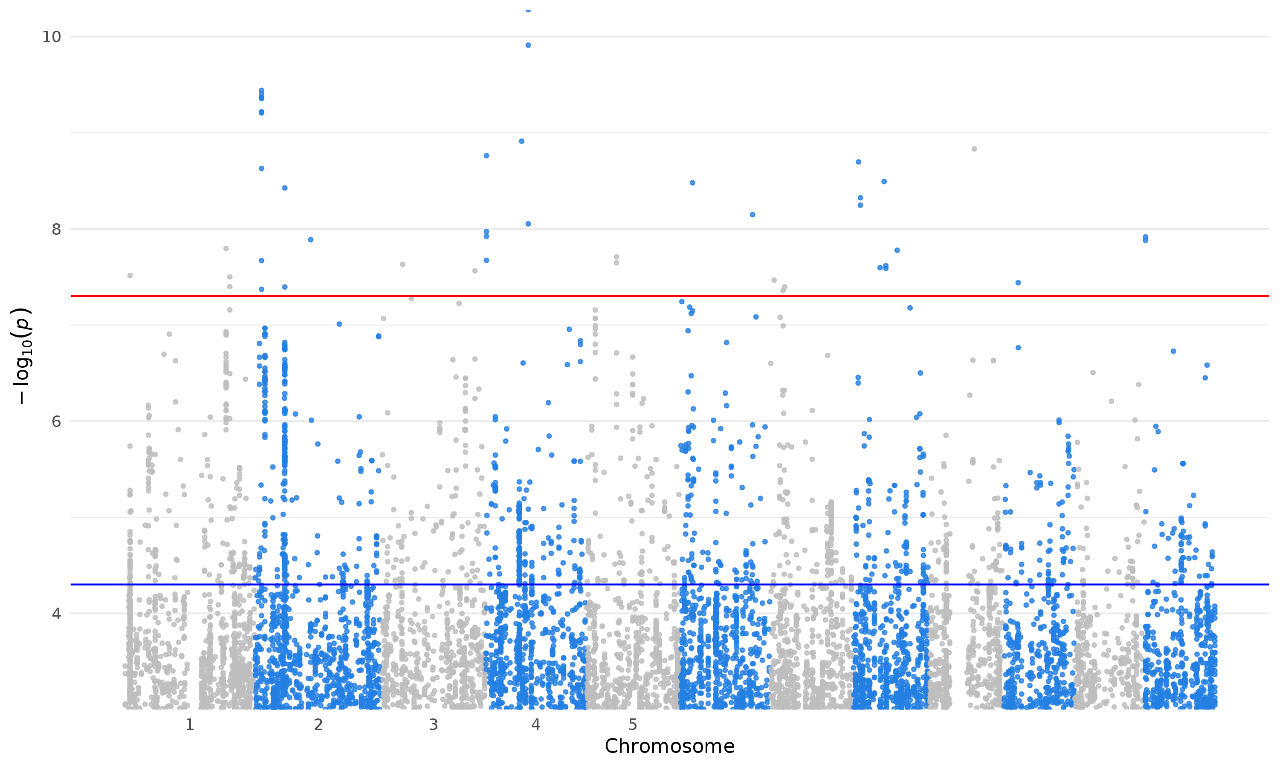


Manhattan plots: 1000 Genomes-based dosage scores (SNPs with R^2^ > 0.3& MAF > 0.02) for the BN


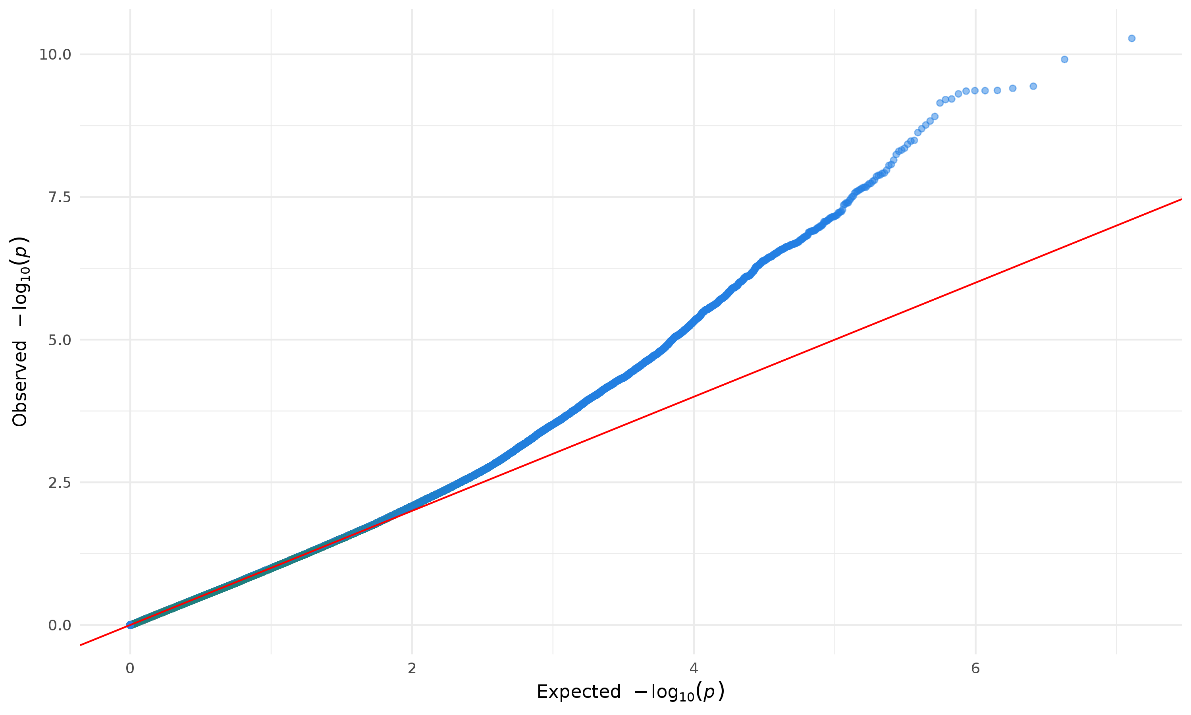


QQ Plot for the BN

**LD Score Regression (LDSC):**

Total Observed scale h2: 0.4385 (0.1898)

Lambda GC: 1.0432

Mean Chi^2: 1.0181

Intercept: 0.9973 (0.007)

Ratio < 0 (usually indicates GC correction).
