# Supplementary material for: Causal relationship between bulimia nervosa and microstructural white matter: evidence from Mendelian randomization
Source: Eat Weight Disord. 2025 May 19;30(1):41. doi: 10.1007/s40519-025-01754-z (PMC12089160; doi:10.1007/s40519-025-01754-z)
Supplement: Supplementary file 3 — Supplementary file3 (PDF 12422 KB) [file 40519_2025_1754_MOESM3_ESM.pdf]

Catalogue

1. MR anlaysis of IDP dMRI TBSS MD Pontine crossing tract || id:ubm-a-245…………………….. 1

2. MR anlaysis of IDP dMRI TBSS MD Medial lemniscus R || id:ubm-a-252………………….. …. 2

3. MR anlaysis of IDP dMRI TBSS L1 Pontine crossing tract || id:ubm-a-341……………...……. 3

4. MR anlaysis of IDP dMRI TBSS L1 Medial lemniscus R || id:ubm-a-348……………………..….. 4

5. MR anlaysis of IDP dMRI TBSS L1 Medial lemniscus L || id:ubm-a-349…………….………….. 5

6. MR anlaysis of IDP dMRI TBSS L1 Superior cerebellar peduncle L || id:ubm-a-353……………. 6

7. MR anlaysis of IDP dMRI TBSS L1 Anterior corona radiata L || id:ubm-a-363……………….…... 7

8. MR anlaysis of IDP dMRI TBSS OD Medial lemniscus R || id:ubm-a-540………….…………….... 8

9. MR anlaysis of IDP dMRI TBSS OD Medial lemniscus L || id:ubm-a-541…………..…………..... 9

10. MR anlaysis of IDP dMRI TBSS OD Cingulum hippocampus R || id:ubm-a-568……………..….... 10

11. MR anlaysis of IDP dMRI TBSS ISOVF Pontine crossing tract || id:ubm-a-581…………….....… 11

12. MR anlaysis of IDP dMRI TBSS ISOVF Medial lemniscus R || id:ubm-a-588………..………… 12

13. MR anlaysis of IDP dMRI TBSS ISOVF Medial lemniscus L || id:ubm-a-589………...………. 13

14. MR anlaysis of IDP dMRI TBSS ISOVF Superior cerebellar peduncle L || id:ubm-a-593………. 14

15. MR anlaysis of IDP dMRI TBSS ISOVF Retrolenticular part of internal capsule R || id:ubm-a-600………………………………………………….…………………………….……...…..……. 15

16. MR anlaysis of IDP dMRI ProbtrackX L1 ml r || id:ubm-a-727……………….………...……….. 16

17. MR anlaysis of IDP dMRI ProbtrackX OD ilf l || id:ubm-a-831……………...………………...….. 17

18. MR anlaysis of IDP dMRI ProbtrackX OD mcp || id:ubm-a-833……………....………...……….. 18

Note: The MR analysis result plots for each IDP show the comparison of results using different MR methods (Figure A), funnel plot (Figure B), forest plot of single SNP MR (Figure C) and leave-one-out sensitivity analysis result (Figure D) for that IDP, respectively.

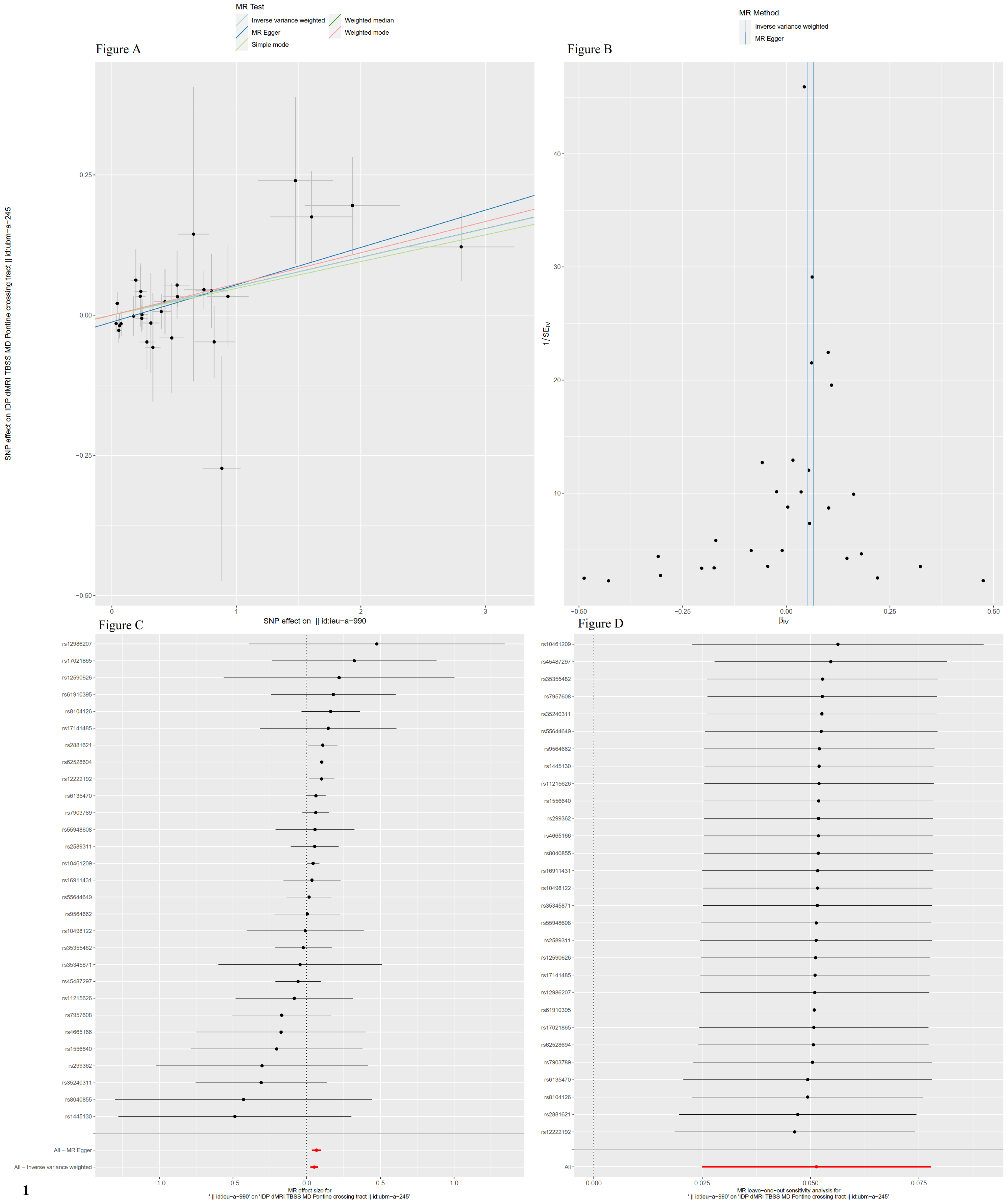
MR Test
Inverse variance weighted
MR Egger
Simple mode
Weighted median
Weighted mode
Figure A
SNP effect on IDP dMRI TBSS MD Pontine crossing tract || id:ubm-a-245
SNP effect on || id:ieu-a-990
MR Method
Inverse variance weighted
MR Egger
Figure B
1/SE$_{IV}$
β_{IV}
Figure C
rs12986207
rs17021865
rs12590626
rs61910395
rs8104126
rs17141485
rs2881621
rs62528694
rs12222192
rs6135470
rs7903789
rs55948608
rs2589311
rs10461209
rs16911431
rs55644649
rs9564662
rs10498122
rs35355482
rs35345871
rs45487297
rs11215626
rs7957608
rs4665166
rs1556640
rs299362
rs35240311
rs8040855
rs1445130
All – MR Egger
All – Inverse variance weighted
MR effect size for
' || id:ieu-a-990' on 'IDP dMRI TBSS MD Pontine crossing tract || id:ubm-a-245'
Figure D
rs10461209
rs45487297
rs35355482
rs7957608
rs35240311
rs55644649
rs9564662
rs1445130
rs11215626
rs1556640
rs299362
rs4665166
rs8040855
rs16911431
rs10498122
rs35345871
rs55948608
rs2589311
rs12590626
rs17141485
rs12986207
rs61910395
rs17021865
rs62528694
rs7903789
rs6135470
rs8104126
rs2881621
rs12222192
All
MR leave–one–out sensitivity analysis for
' || id:ieu-a-990' on 'IDP dMRI TBSS MD Pontine crossing tract || id:ubm-a-245'

1

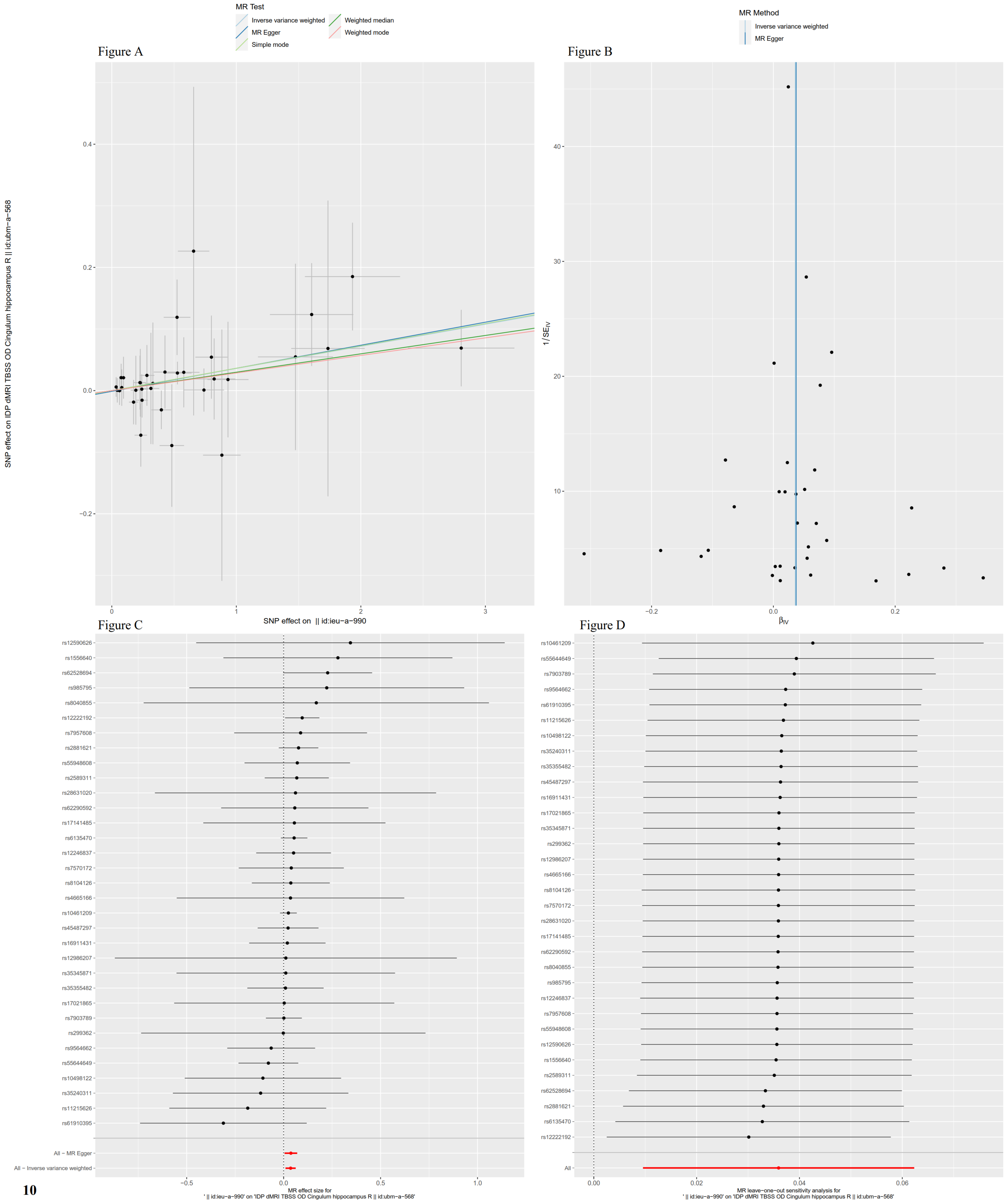

MR Test
Inverse variance weighted
MR Egger
Simple mode
Weighted median
Weighted mode
Figure A
SNP effect on IDP dMRI TBSS OD Cingulum hippocampus R || id:ubm-a-568
SNP effect on || id:ieu-a-990
MR Method
Inverse variance weighted
MR Egger
Figure B
1/SE_IV
β_IV
Figure C
rs12590626
rs1556640
rs62528694
rs985795
rs8040855
rs12222192
rs7957608
rs2881621
rs55948608
rs2589311
rs28631020
rs62290592
rs17141485
rs6135470
rs12246837
rs7570172
rs8104126
rs4665166
rs10461209
rs45487297
rs16911431
rs12986207
rs35345871
rs35355482
rs17021865
rs7903789
rs299362
rs9564662
rs55644649
rs10498122
rs35240311
rs11215626
rs61910395
All − MR Egger
All − Inverse variance weighted
MR effect size for
' || id:ieu−a−990' on 'IDP dMRI TBSS OD Cingulum hippocampus R || id:ubm−a−568'
Figure D
rs10461209
rs55644649
rs7903789
rs9564662
rs61910395
rs11215626
rs10498122
rs35240311
rs35355482
rs45487297
rs16911431
rs17021865
rs35345871
rs299362
rs12986207
rs4665166
rs8104126
rs7570172
rs28631020
rs17141485
rs62290592
rs8040855
rs985795
rs12246837
rs7957608
rs55948608
rs12590626
rs1556640
rs2589311
rs62528694
rs2881621
rs6135470
rs12222192
All
MR leave−one−out sensitivity analysis for
' || id:ieu−a−990' on 'IDP dMRI TBSS OD Cingulum hippocampus R || id:ubm−a−568'

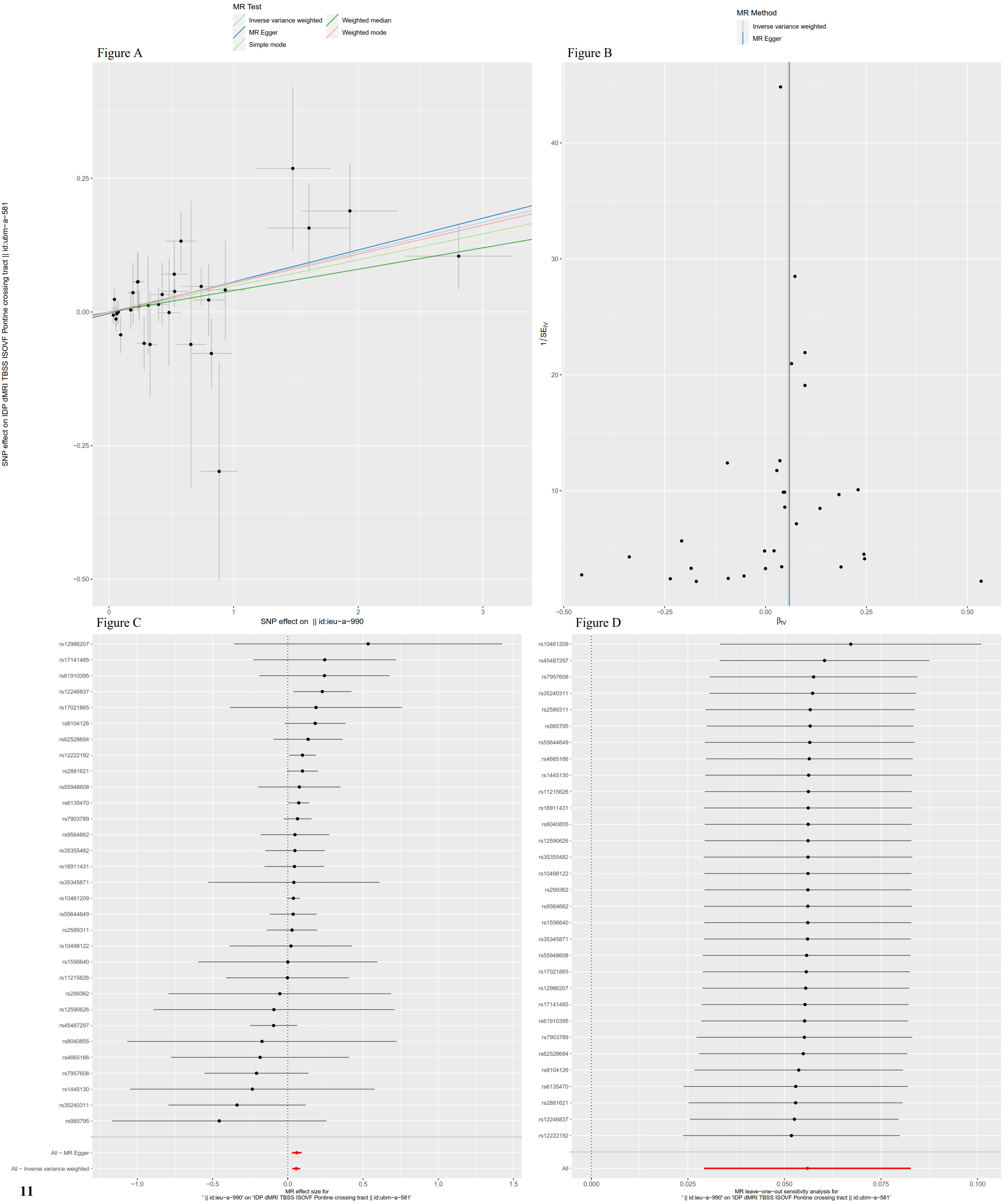
MR Test
Inverse variance weighted
MR Egger
Simple mode
Weighted median
Weighted mode
MR Method
Inverse variance weighted
MR Egger
Figure A
SNP effect on IDP dMRI TBSS ISOVF Pontine crossing tract || id:ubm−a−581
SNP effect on || id:ieu−a−990
Figure B
1/SE_IV
β_IV
Figure C
rs12986207
rs17141485
rs61910395
rs12246837
rs17021865
rs8104126
rs62528694
rs12222192
rs2881621
rs55948608
rs6135470
rs7903789
rs9564662
rs35355482
rs16911431
rs35345871
rs10461209
rs55644649
rs2589311
rs10498122
rs1556640
rs11215626
rs299362
rs12590626
rs45487297
rs8040855
rs4665166
rs7957608
rs1445130
rs35240311
rs985795
All − MR Egger
All − Inverse variance weighted
MR effect size for
' || id:ieu−a−990' on 'IDP dMRI TBSS ISOVF Pontine crossing tract || id:ubm−a−581'
Figure D
rs10461209
rs45487297
rs7957608
rs35240311
rs2589311
rs985795
rs55644649
rs4665166
rs1445130
rs11215626
rs16911431
rs8040855
rs12590626
rs35355482
rs10498122
rs299362
rs9564662
rs1556640
rs35345871
rs55948608
rs17021865
rs12986207
rs17141485
rs61910395
rs7903789
rs62528694
rs8104126
rs6135470
rs2881621
rs12246837
rs12222192
All
MR leave−one−out sensitivity analysis for
' || id:ieu−a−990' on 'IDP dMRI TBSS ISOVF Pontine crossing tract || id:ubm−a−581'

Figure A

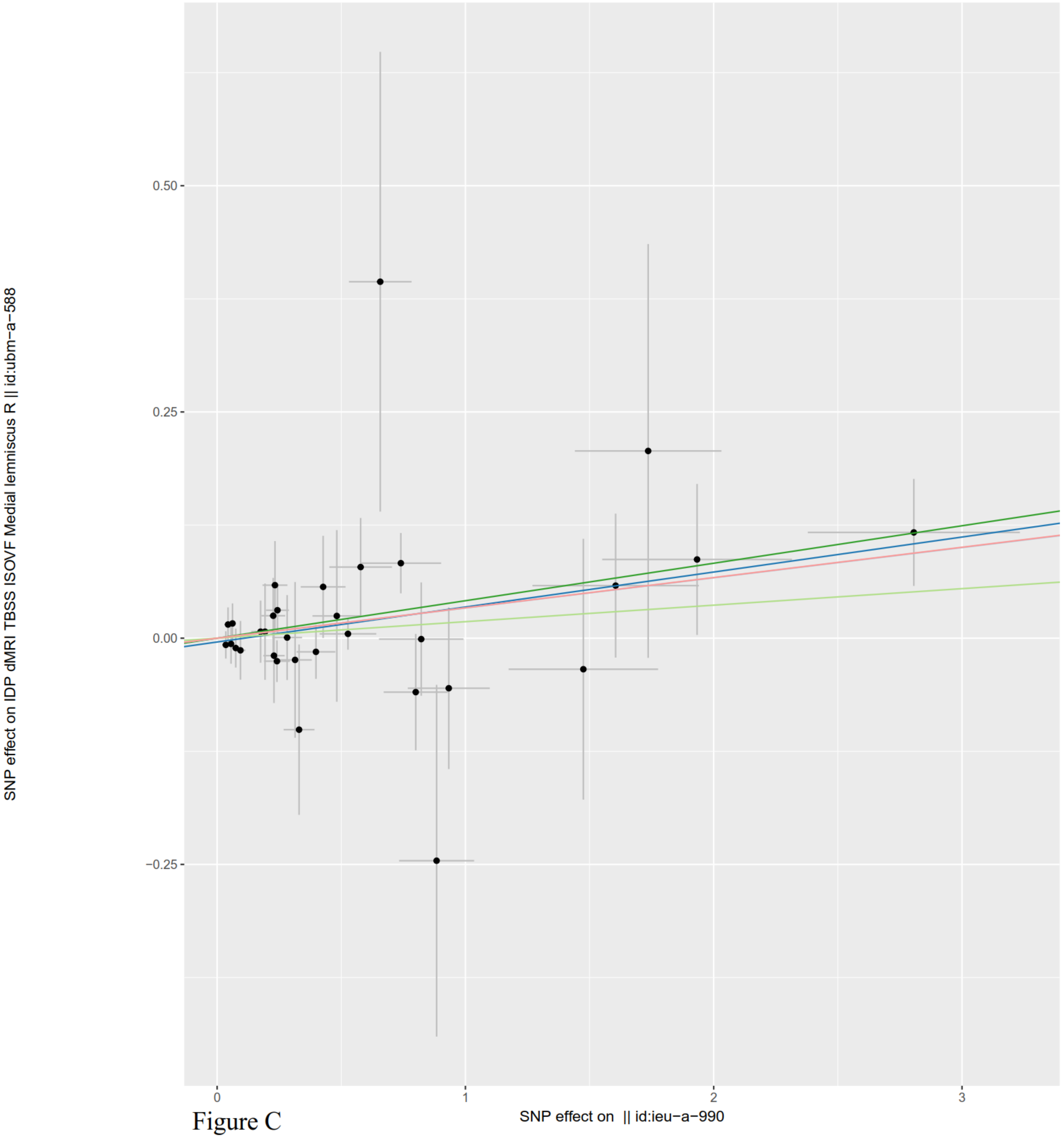

Figure B

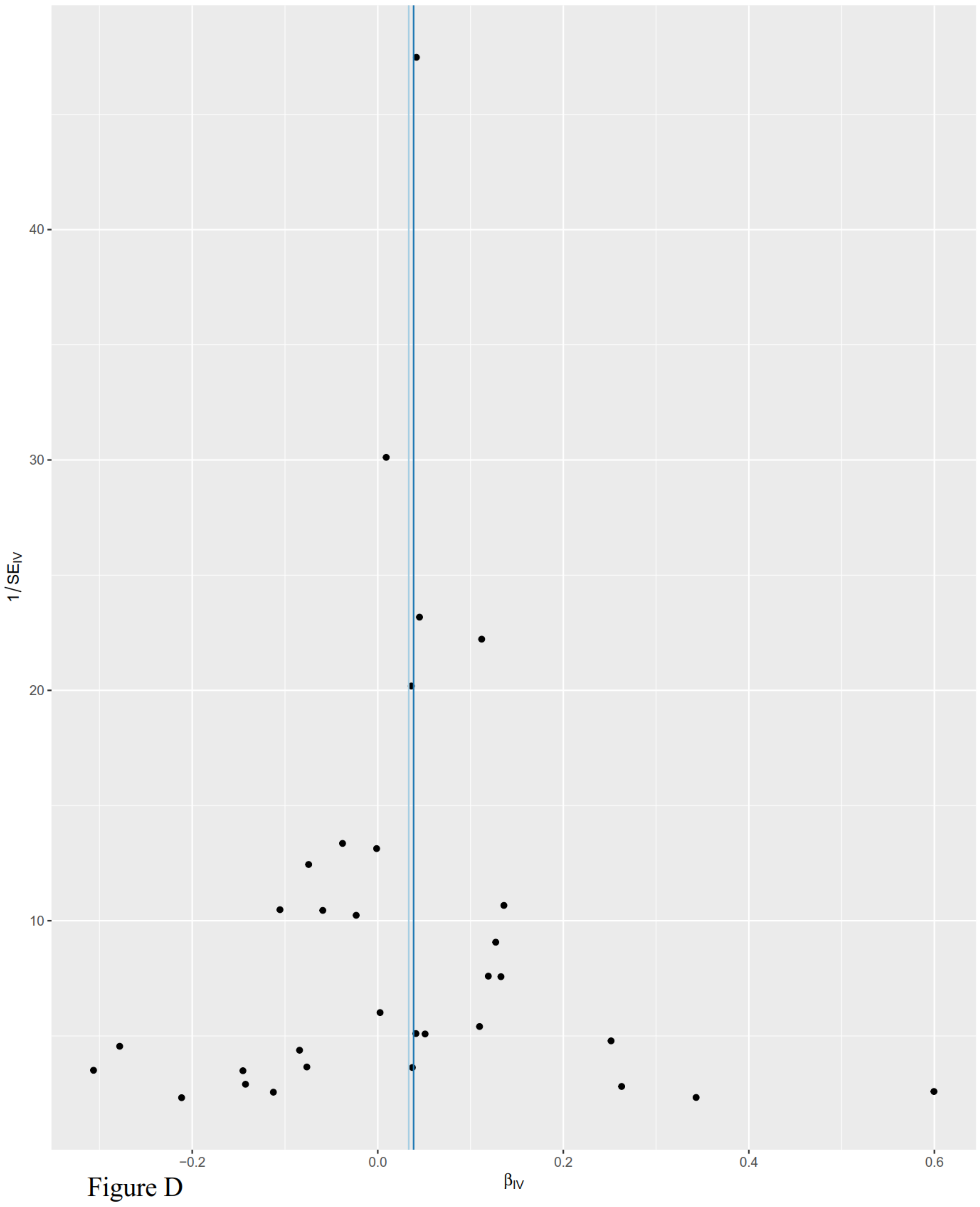

Figure C

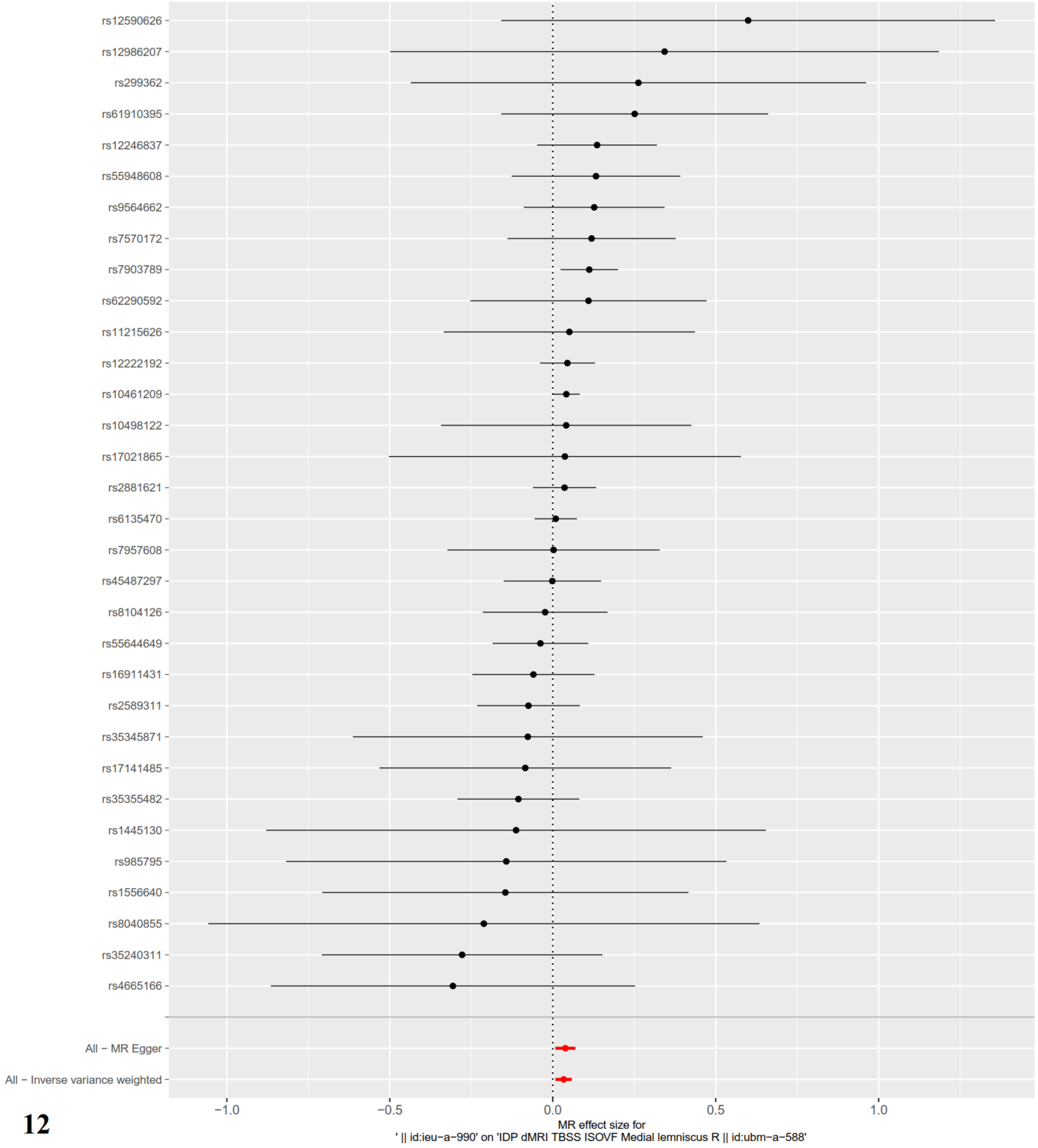

Figure D

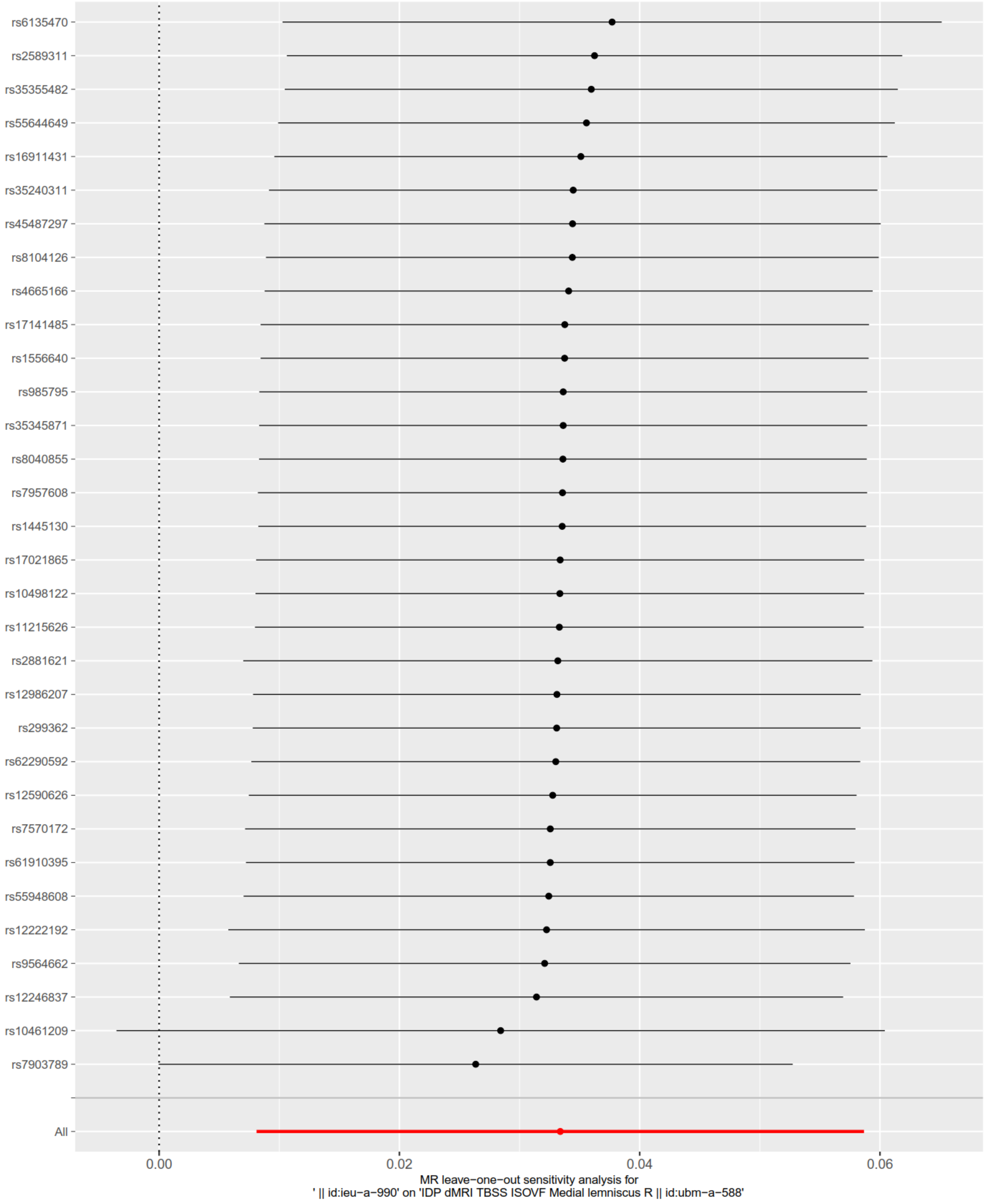

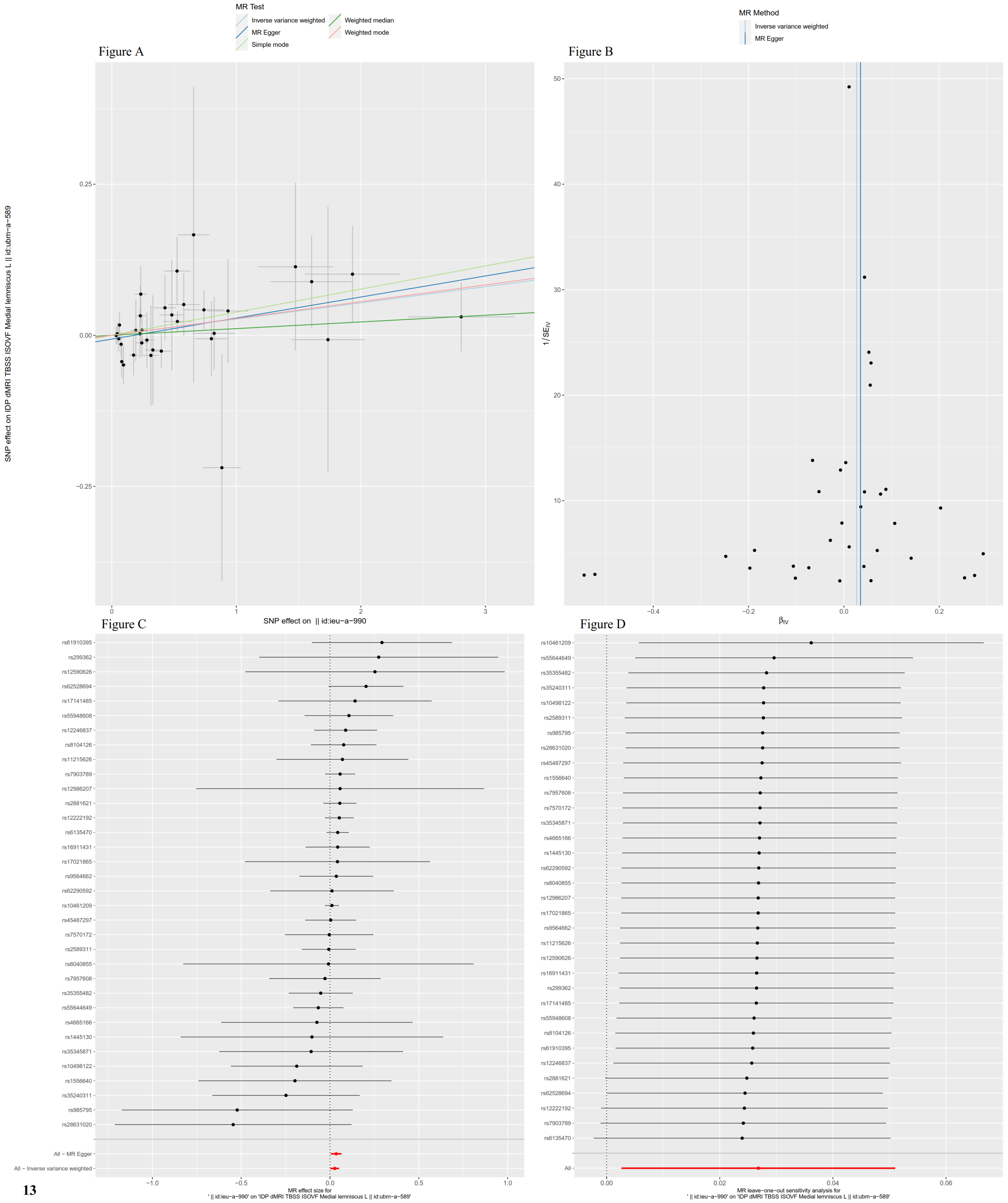

Figure A
MR Test
Inverse variance weighted
MR Egger
Simple mode
Weighted median
Weighted mode
SNP effect on IDP dMRI TBSS ISOVF Medial lemniscus L || id:ubm−a−589
SNP effect on || id:ieu−a−990
Figure B
MR Method
Inverse variance weighted
MR Egger
$1/SE_{IV}$
β_{IV}
Figure C
rs61910395
rs299362
rs12590626
rs62528694
rs17141485
rs55948608
rs12246837
rs8104126
rs11215626
rs7903789
rs12986207
rs2881621
rs12222192
rs6135470
rs16911431
rs17021865
rs9564662
rs62290592
rs10461209
rs45487297
rs7570172
rs2589311
rs8040855
rs7957608
rs35355482
rs55644649
rs4665166
rs1445130
rs35345871
rs10498122
rs1556640
rs35240311
rs985795
rs28631020
All − MR Egger
All − Inverse variance weighted
MR effect size for
' || id:ieu−a−990' on 'IDP dMRI TBSS ISOVF Medial lemniscus L || id:ubm−a−589'
Figure D
rs10461209
rs55644649
rs35355482
rs35240311
rs10498122
rs2589311
rs985795
rs28631020
rs45487297
rs1556640
rs7957608
rs7570172
rs35345871
rs4665166
rs1445130
rs62290592
rs8040855
rs12986207
rs17021865
rs9564662
rs11215626
rs12590626
rs16911431
rs299362
rs17141485
rs55948608
rs8104126
rs61910395
rs12246837
rs2881621
rs62528694
rs12222192
rs7903789
rs6135470
All
MR leave−one−out sensitivity analysis for
' || id:ieu−a−990' on 'IDP dMRI TBSS ISOVF Medial lemniscus L || id:ubm−a−589'

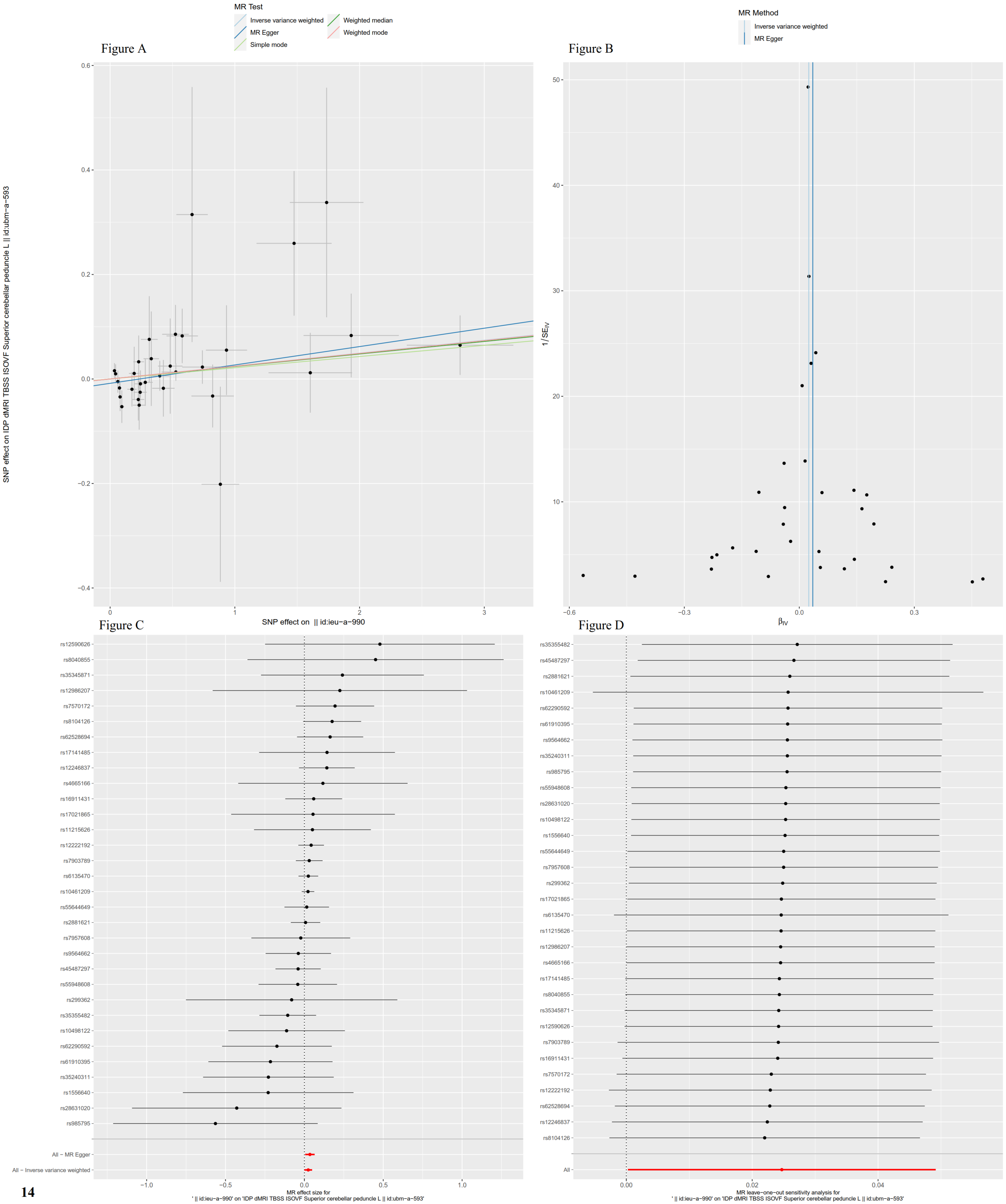
Figure A
MR Test
Inverse variance weighted
MR Egger
Simple mode
Weighted median
Weighted mode
SNP effect on IDP dMRI TBSS ISOVF Superior cerebellar peduncle L || id:ubm−a−593
SNP effect on || id:ieu−a−990
Figure B
MR Method
Inverse variance weighted
MR Egger
$1/SE_{IV}$
β_{IV}
Figure C
rs12590626
rs8040855
rs35345871
rs12986207
rs7570172
rs8104126
rs62528694
rs17141485
rs12246837
rs4665166
rs16911431
rs17021865
rs11215626
rs12222192
rs7903789
rs6135470
rs10461209
rs55644649
rs2881621
rs7957608
rs9564662
rs45487297
rs55948608
rs299362
rs35355482
rs10498122
rs62290592
rs61910395
rs35240311
rs1556640
rs28631020
rs985795
All − MR Egger
All − Inverse variance weighted
MR effect size for
' || id:ieu−a−990' on 'IDP dMRI TBSS ISOVF Superior cerebellar peduncle L || id:ubm−a−593'
Figure D
rs35355482
rs45487297
rs2881621
rs10461209
rs62290592
rs61910395
rs9564662
rs35240311
rs985795
rs55948608
rs28631020
rs10498122
rs1556640
rs55644649
rs7957608
rs299362
rs17021865
rs6135470
rs11215626
rs12986207
rs4665166
rs17141485
rs8040855
rs35345871
rs12590626
rs7903789
rs16911431
rs7570172
rs12222192
rs62528694
rs12246837
rs8104126
All
MR leave−one−out sensitivity analysis for
' || id:ieu−a−990' on 'IDP dMRI TBSS ISOVF Superior cerebellar peduncle L || id:ubm−a−593'

Figure A

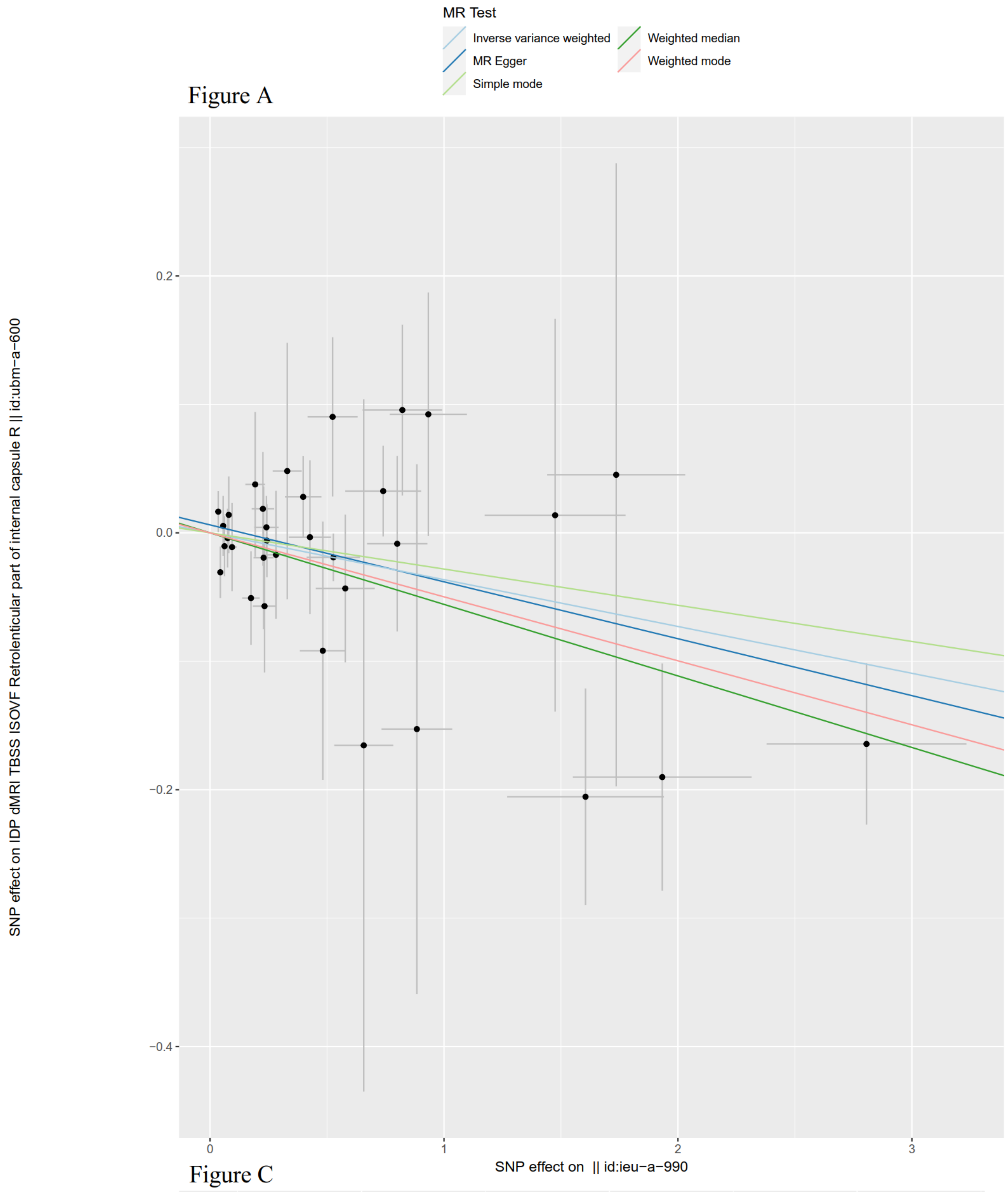

Figure B

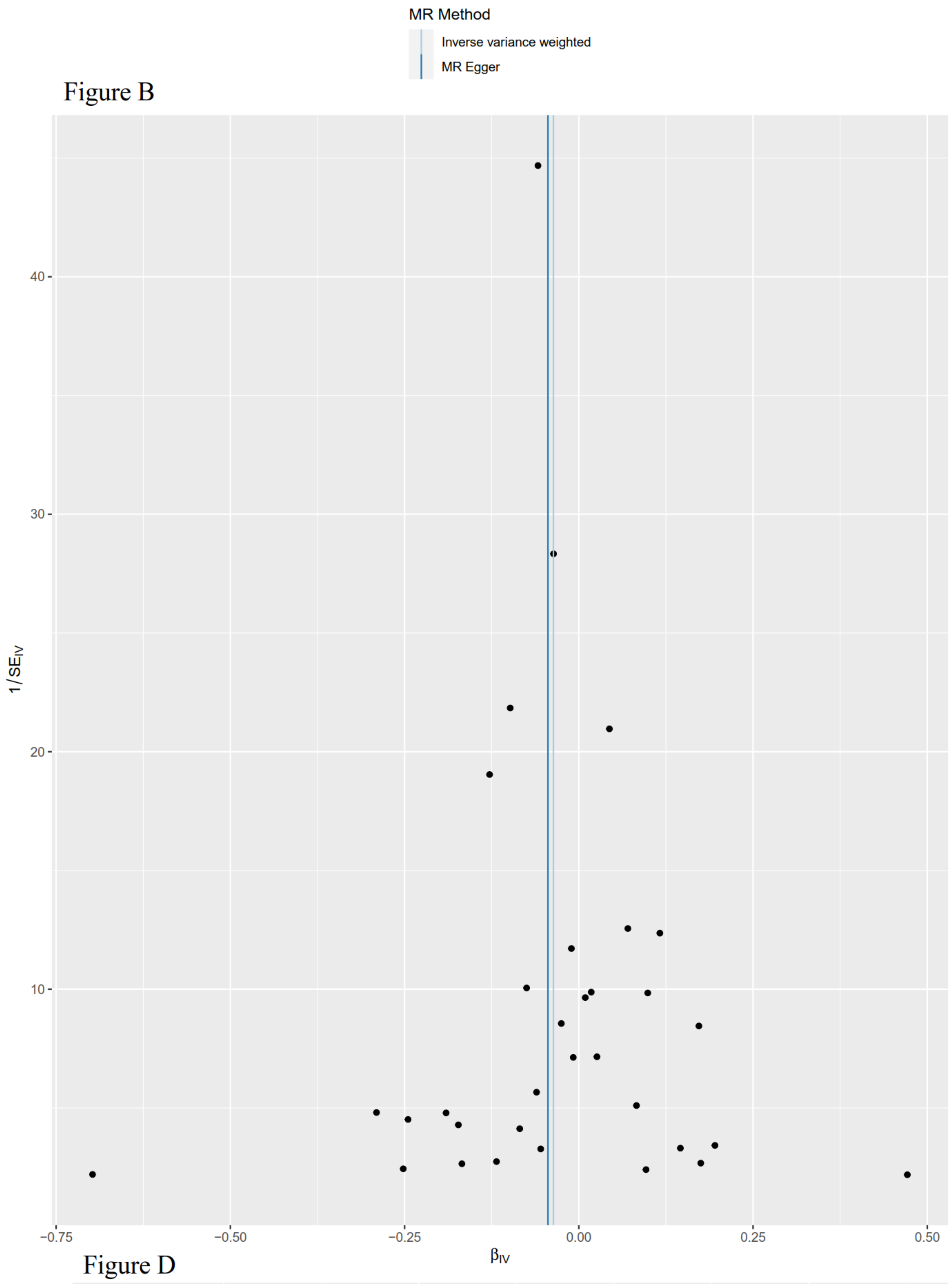

Figure C

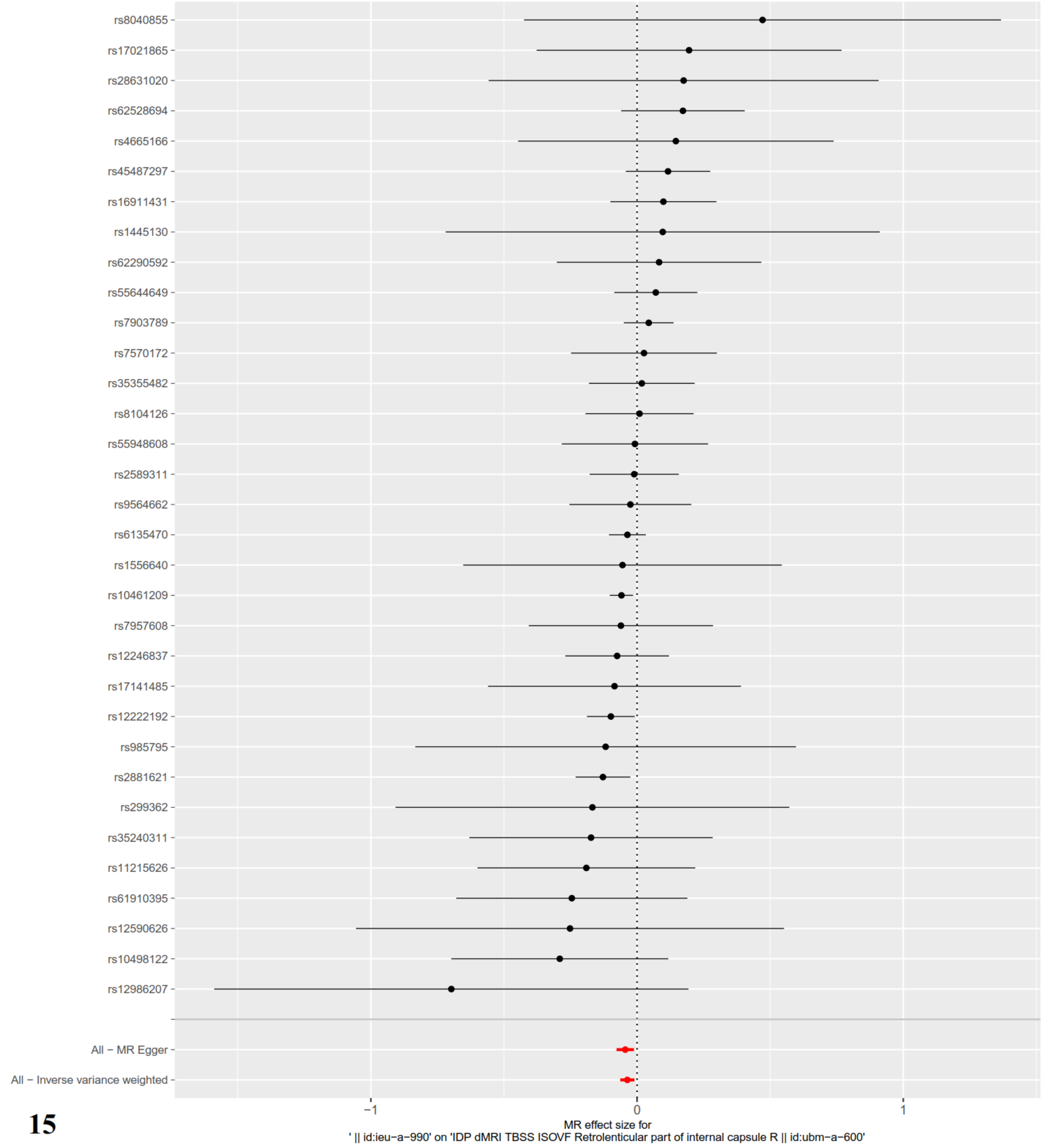

Figure D

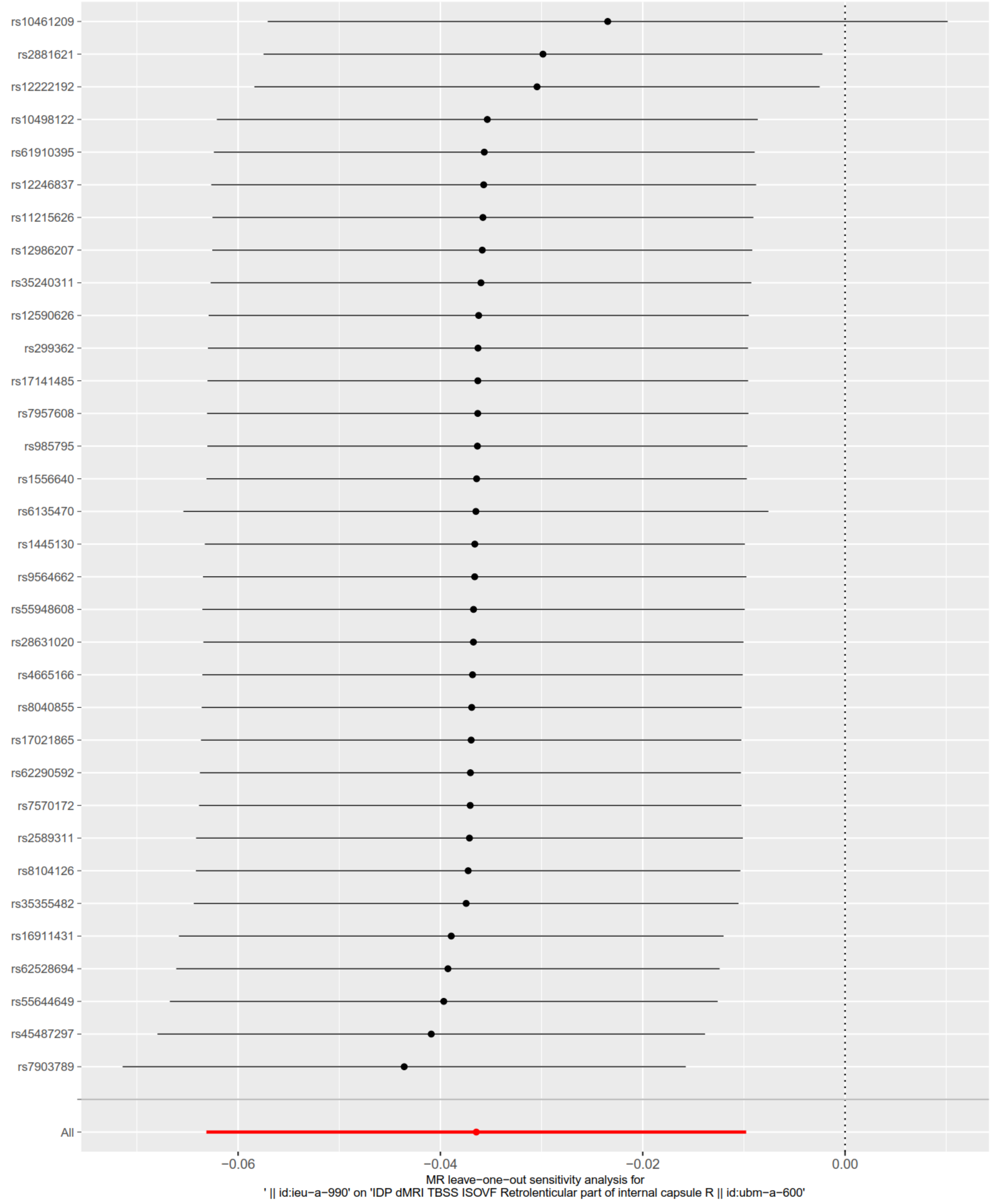

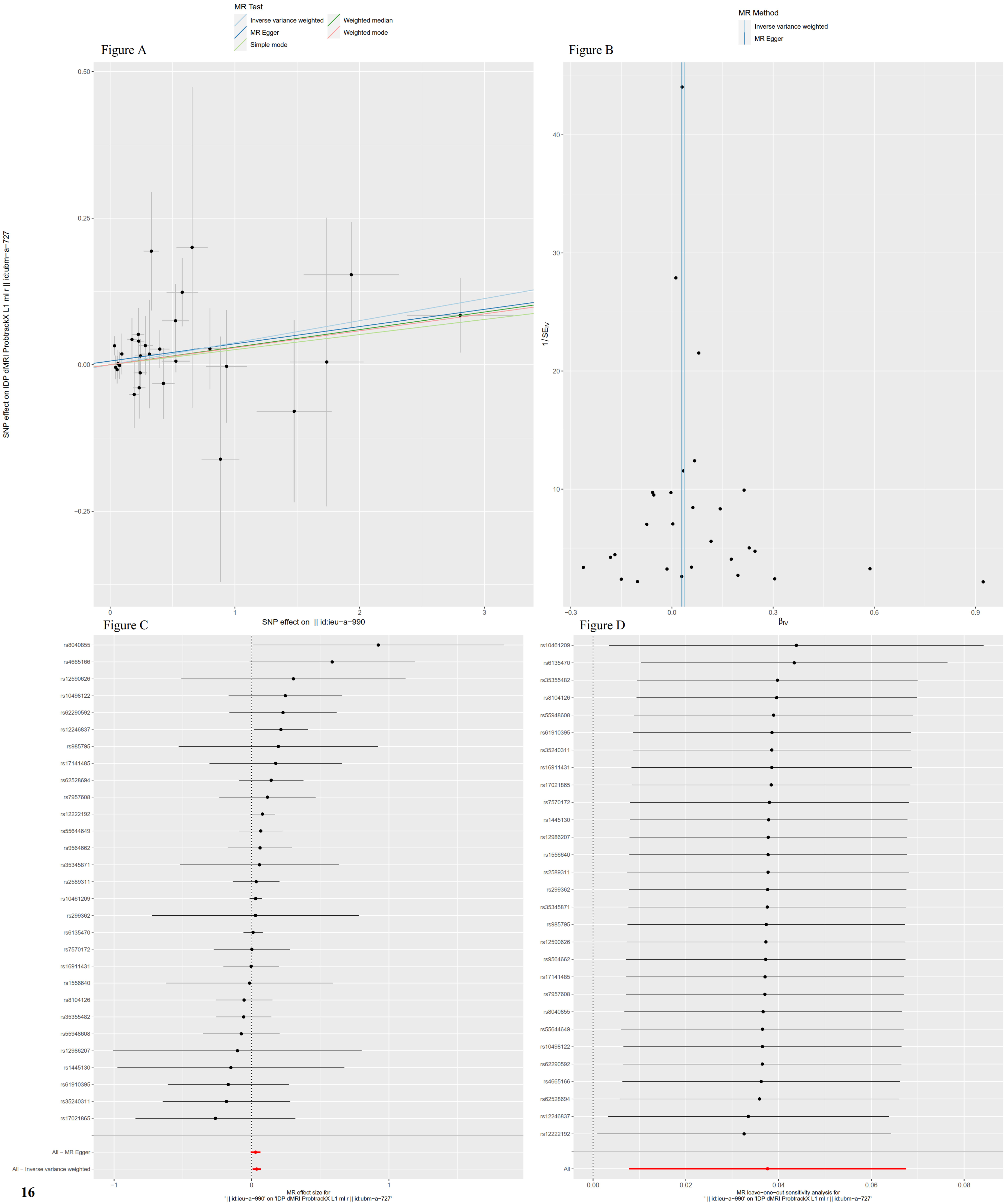

MR Test
Inverse variance weighted
MR Egger
Simple mode
Weighted median
Weighted mode
Figure A
SNP effect on IDP dMRI ProbtrackX L1 ml r || id:ubm−a−727
SNP effect on || id:ieu−a−990
MR Method
Inverse variance weighted
MR Egger
Figure B
1/SE$_{IV}$
β$_{IV}$
Figure C
rs8040855
rs4665166
rs12590626
rs10498122
rs62290592
rs12246837
rs985795
rs17141485
rs62528694
rs7957608
rs12222192
rs55644649
rs9564662
rs35345871
rs2589311
rs10461209
rs299362
rs6135470
rs7570172
rs16911431
rs1556640
rs8104126
rs35355482
rs55948608
rs12986207
rs1445130
rs61910395
rs35240311
rs17021865
All − MR Egger
All − Inverse variance weighted
MR effect size for
' || id:ieu−a−990' on 'IDP dMRI ProbtrackX L1 ml r || id:ubm−a−727'
Figure D
rs10461209
rs6135470
rs35355482
rs8104126
rs55948608
rs61910395
rs35240311
rs16911431
rs17021865
rs7570172
rs1445130
rs12986207
rs1556640
rs2589311
rs299362
rs35345871
rs985795
rs12590626
rs9564662
rs17141485
rs7957608
rs8040855
rs55644649
rs10498122
rs62290592
rs4665166
rs62528694
rs12246837
rs12222192
All
MR leave−one−out sensitivity analysis for
' || id:ieu−a−990' on 'IDP dMRI ProbtrackX L1 ml r || id:ubm−a−727'

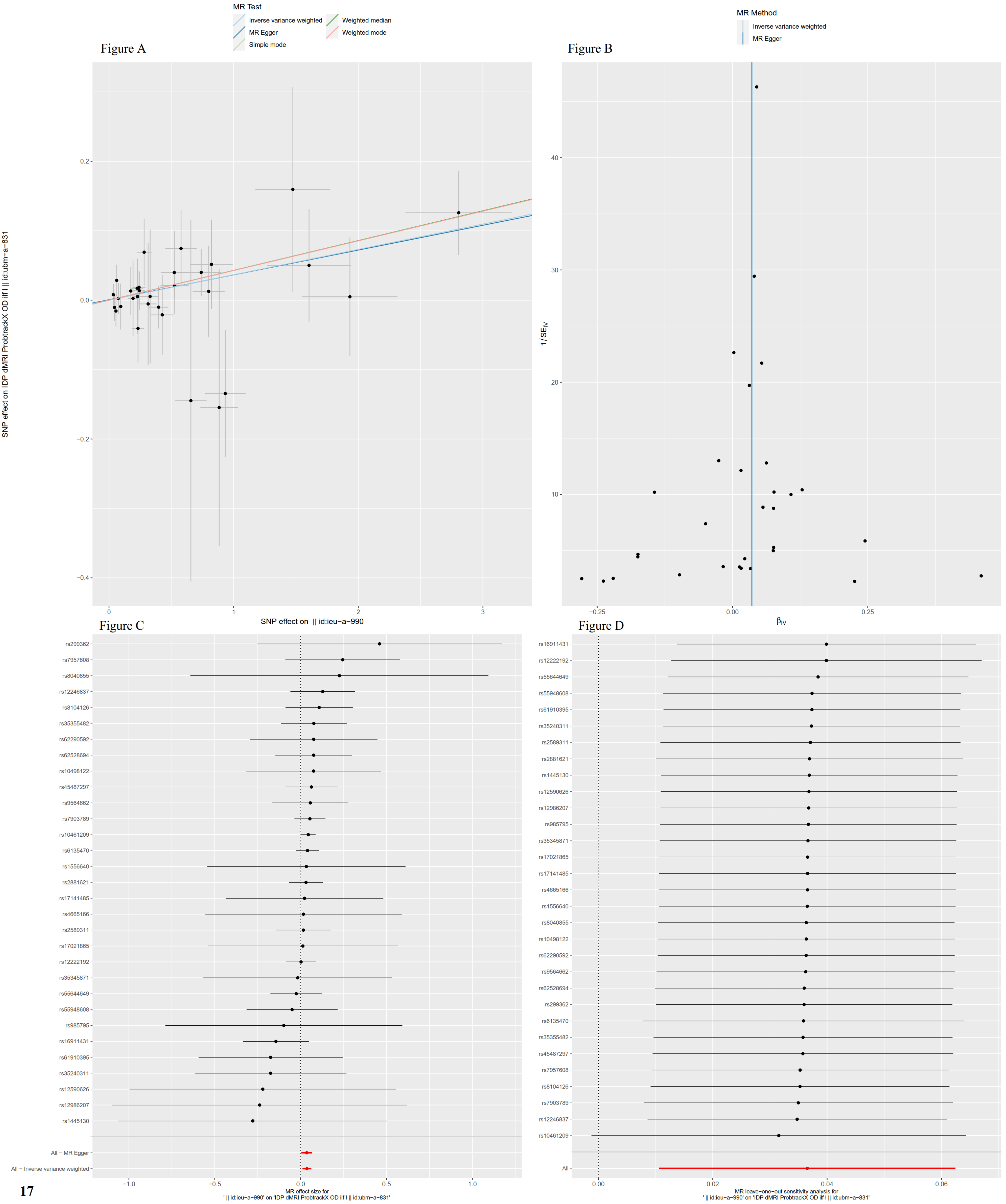

MR Test
Inverse variance weighted
MR Egger
Simple mode
Weighted median
Weighted mode
Figure A
SNP effect on IDP dMRI ProbtrackX OD ilf l || id:ubm-a-831
SNP effect on || id:ieu-a-990
MR Method
Inverse variance weighted
MR Egger
Figure B
1/SE_IV
β_IV
Figure C
rs299362
rs7957608
rs8040855
rs12246837
rs8104126
rs35355482
rs62290592
rs62528694
rs10498122
rs45487297
rs9564662
rs7903789
rs10461209
rs6135470
rs1556640
rs2881621
rs17141485
rs4665166
rs2589311
rs17021865
rs12222192
rs35345871
rs55644649
rs55948608
rs985795
rs16911431
rs61910395
rs35240311
rs12590626
rs12986207
rs1445130
All − MR Egger
All − Inverse variance weighted
MR effect size for
' || id:ieu-a-990' on 'IDP dMRI ProbtrackX OD ilf l || id:ubm-a-831'
Figure D
rs16911431
rs12222192
rs55644649
rs55948608
rs61910395
rs35240311
rs2589311
rs2881621
rs1445130
rs12590626
rs12986207
rs985795
rs35345871
rs17021865
rs17141485
rs4665166
rs1556640
rs8040855
rs10498122
rs62290592
rs9564662
rs62528694
rs299362
rs6135470
rs35355482
rs45487297
rs7957608
rs8104126
rs7903789
rs12246837
rs10461209
All
MR leave−one−out sensitivity analysis for
' || id:ieu-a-990' on 'IDP dMRI ProbtrackX OD ilf l || id:ubm-a-831'

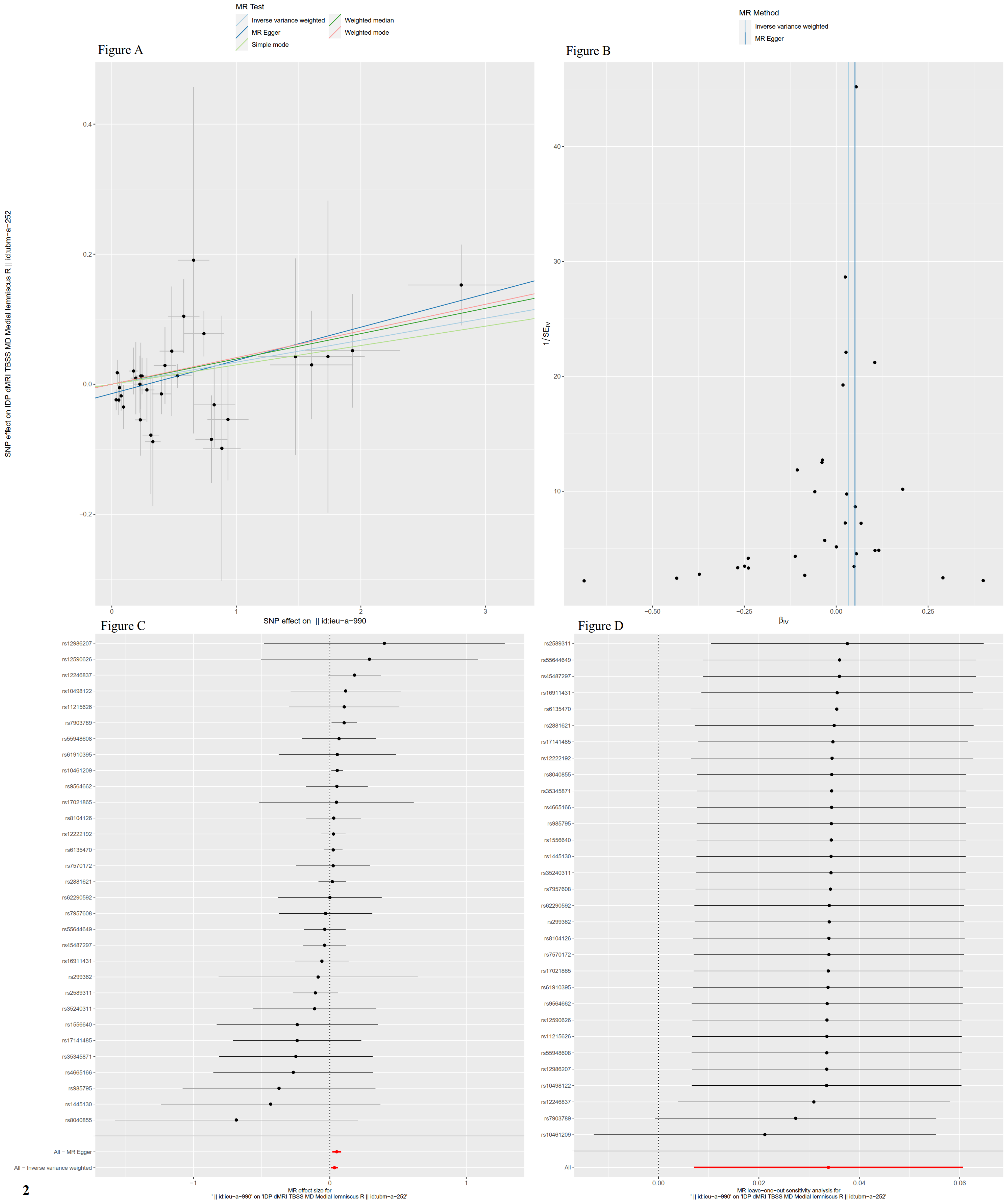

MR Test
Inverse variance weighted
MR Egger
Simple mode
Weighted median
Weighted mode
Figure A
SNP effect on IDP dMRI TBSS MD Medial lemniscus R || id:ubm−a−252
SNP effect on || id:ieu−a−990
MR Method
Inverse variance weighted
MR Egger
Figure B
1/SE$_{IV}$
β$_{IV}$
Figure C
rs12986207
rs12590626
rs12246837
rs10498122
rs11215626
rs7903789
rs55948608
rs61910395
rs10461209
rs9564662
rs17021865
rs8104126
rs12222192
rs6135470
rs7570172
rs2881621
rs62290592
rs7957608
rs55644649
rs45487297
rs16911431
rs299362
rs2589311
rs35240311
rs1556640
rs17141485
rs35345871
rs4665166
rs985795
rs1445130
rs8040855
All − MR Egger
All − Inverse variance weighted
MR effect size for
' || id:ieu−a−990' on 'IDP dMRI TBSS MD Medial lemniscus R || id:ubm−a−252'
Figure D
rs2589311
rs55644649
rs45487297
rs16911431
rs6135470
rs2881621
rs17141485
rs12222192
rs8040855
rs35345871
rs4665166
rs985795
rs1556640
rs1445130
rs35240311
rs7957608
rs62290592
rs299362
rs8104126
rs7570172
rs17021865
rs61910395
rs9564662
rs12590626
rs11215626
rs55948608
rs12986207
rs10498122
rs12246837
rs7903789
rs10461209
All
MR leave−one−out sensitivity analysis for
' || id:ieu−a−990' on 'IDP dMRI TBSS MD Medial lemniscus R || id:ubm−a−252'

2

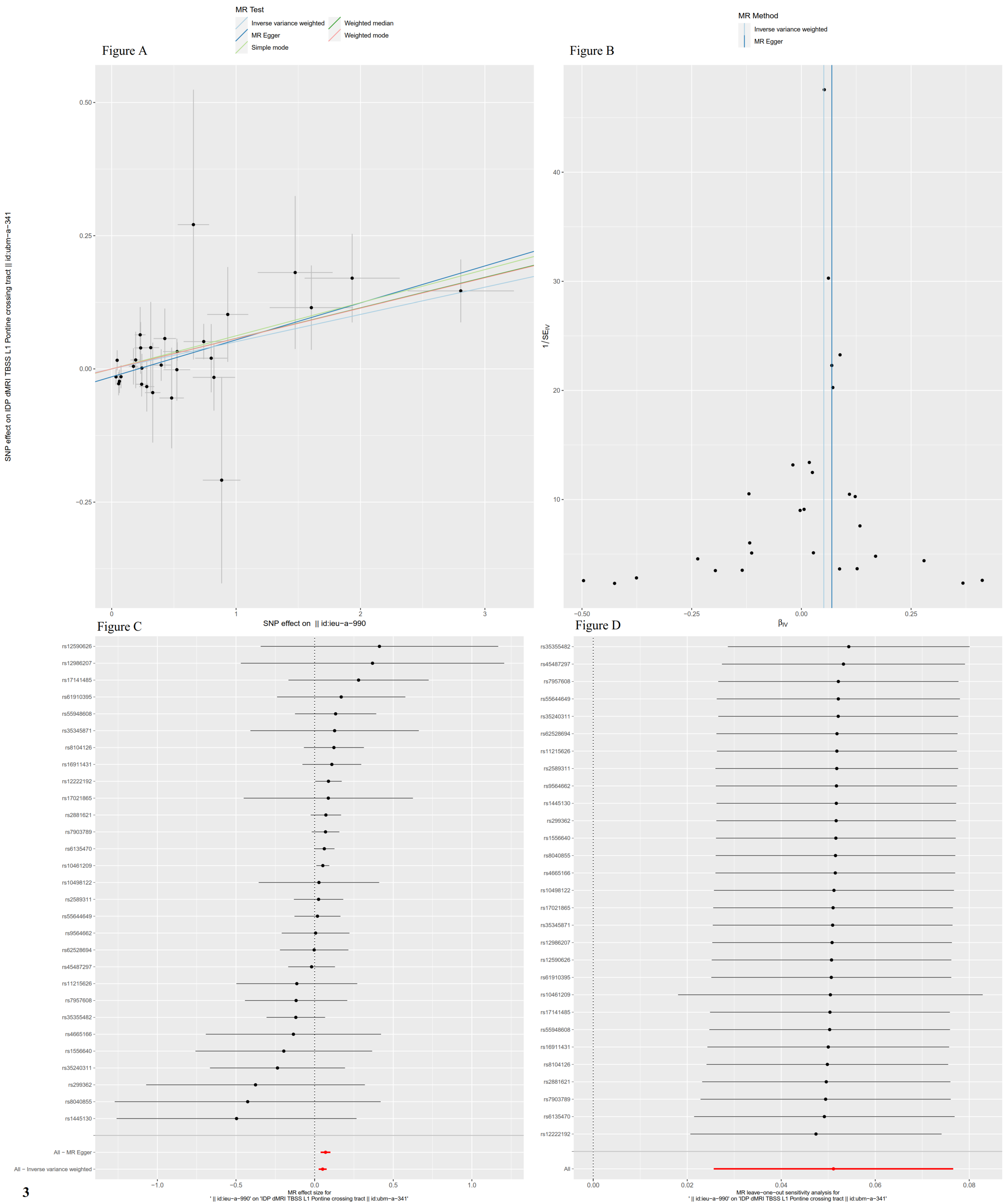

MR Test
Inverse variance weighted
MR Egger
Simple mode
Weighted median
Weighted mode
Figure A
SNP effect on IDP dMRI TBSS L1 Pontine crossing tract || id:ubm-a-341
SNP effect on || id:ieu-a-990
MR Method
Inverse variance weighted
MR Egger
Figure B
1/SE$_{IV}$
β_{IV}
Figure C
rs12590626
rs12986207
rs17141485
rs61910395
rs55948608
rs35345871
rs8104126
rs16911431
rs12222192
rs17021865
rs2881621
rs7903789
rs6135470
rs10461209
rs10498122
rs2589311
rs55644649
rs9564662
rs62528694
rs45487297
rs11215626
rs7957608
rs35355482
rs4665166
rs1556640
rs35240311
rs299362
rs8040855
rs1445130
All − MR Egger
All − Inverse variance weighted
MR effect size for
' || id:ieu-a-990' on 'IDP dMRI TBSS L1 Pontine crossing tract || id:ubm-a-341'
Figure D
rs35355482
rs45487297
rs7957608
rs55644649
rs35240311
rs62528694
rs11215626
rs2589311
rs9564662
rs1445130
rs299362
rs1556640
rs8040855
rs4665166
rs10498122
rs17021865
rs35345871
rs12986207
rs12590626
rs61910395
rs10461209
rs17141485
rs55948608
rs16911431
rs8104126
rs2881621
rs7903789
rs6135470
rs12222192
All
MR leave−one−out sensitivity analysis for
' || id:ieu-a-990' on 'IDP dMRI TBSS L1 Pontine crossing tract || id:ubm-a-341'

3

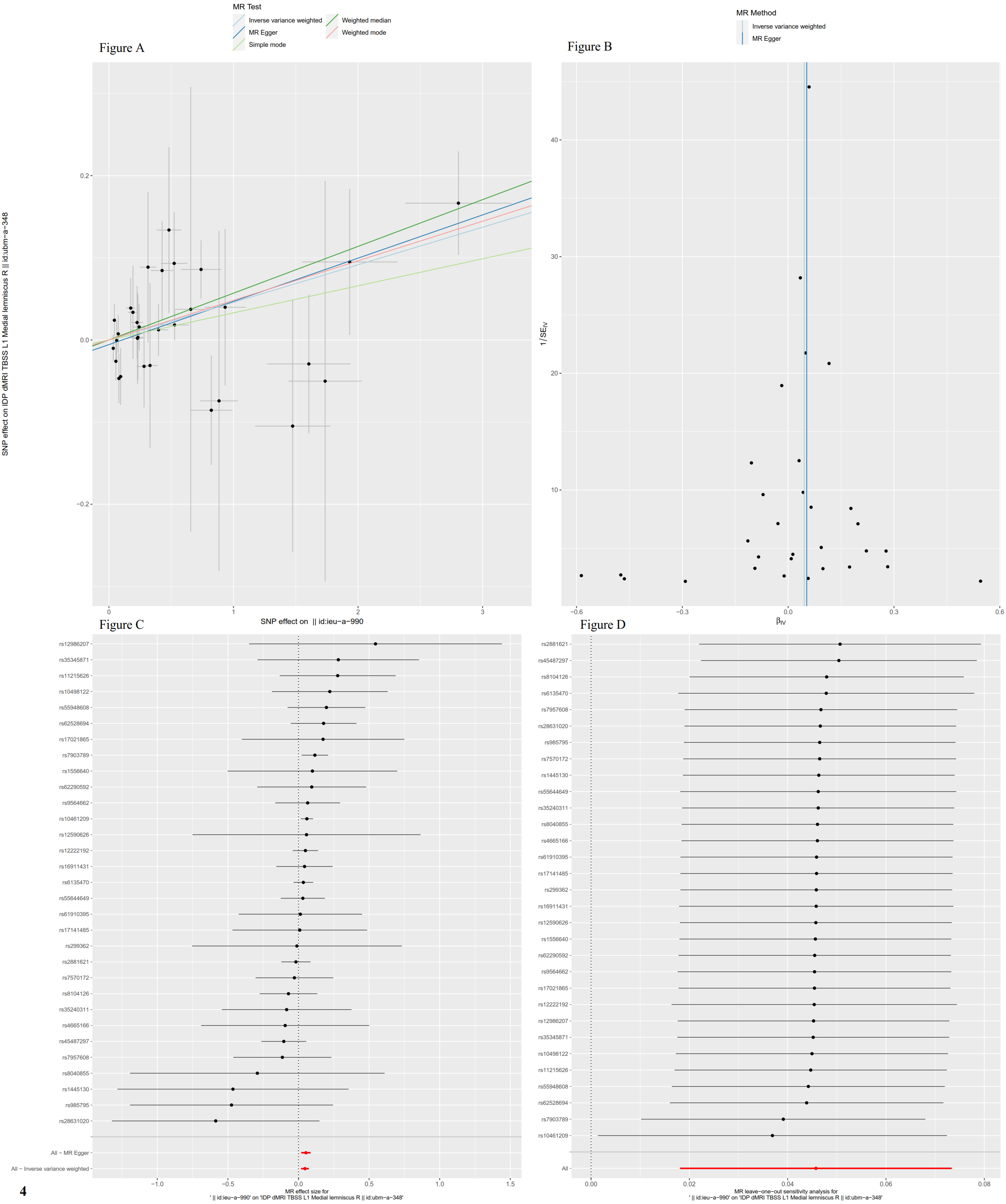
Figure A
MR Test
Inverse variance weighted
MR Egger
Simple mode
Weighted median
Weighted mode
SNP effect on IDP dMRI TBSS L1 Medial lemniscus R || id:ubm−a−348
SNP effect on || id:ieu−a−990
Figure B
MR Method
Inverse variance weighted
MR Egger
1/SEIV
βIV
Figure C
rs12986207
rs35345871
rs11215626
rs10498122
rs55948608
rs62528694
rs17021865
rs7903789
rs1556640
rs62290592
rs9564662
rs10461209
rs12590626
rs12222192
rs16911431
rs6135470
rs55644649
rs61910395
rs17141485
rs299362
rs2881621
rs7570172
rs8104126
rs35240311
rs4665166
rs45487297
rs7957608
rs8040855
rs1445130
rs985795
rs28631020
All − MR Egger
All − Inverse variance weighted
MR effect size for
' || id:ieu−a−990' on 'IDP dMRI TBSS L1 Medial lemniscus R || id:ubm−a−348'
Figure D
rs2881621
rs45487297
rs8104126
rs6135470
rs7957608
rs28631020
rs985795
rs7570172
rs1445130
rs55644649
rs35240311
rs8040855
rs4665166
rs61910395
rs17141485
rs299362
rs16911431
rs12590626
rs1556640
rs62290592
rs9564662
rs17021865
rs12222192
rs12986207
rs35345871
rs10498122
rs11215626
rs55948608
rs62528694
rs7903789
rs10461209
All
MR leave−one−out sensitivity analysis for
' || id:ieu−a−990' on 'IDP dMRI TBSS L1 Medial lemniscus R || id:ubm−a−348'

Figure A

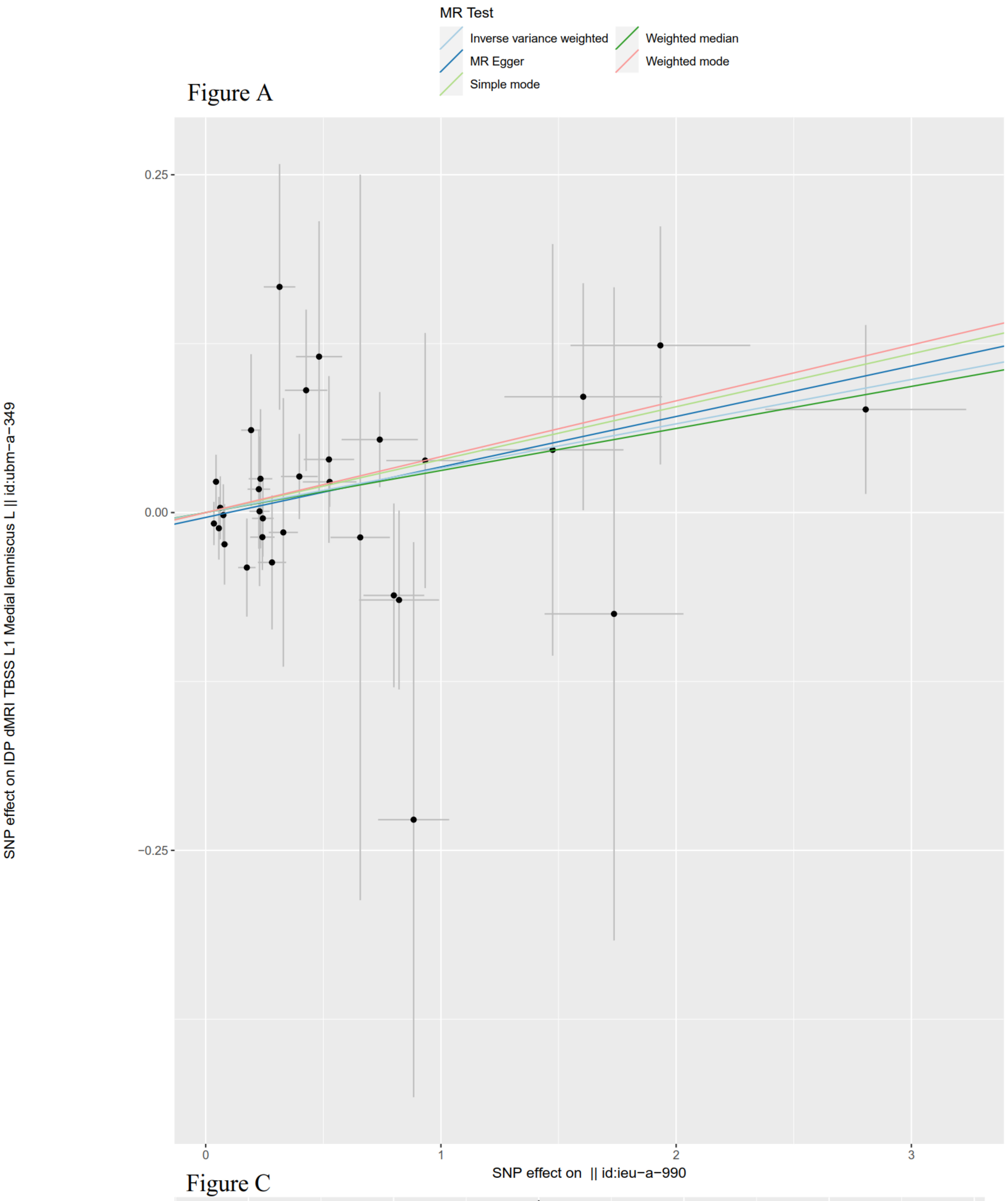

Figure B

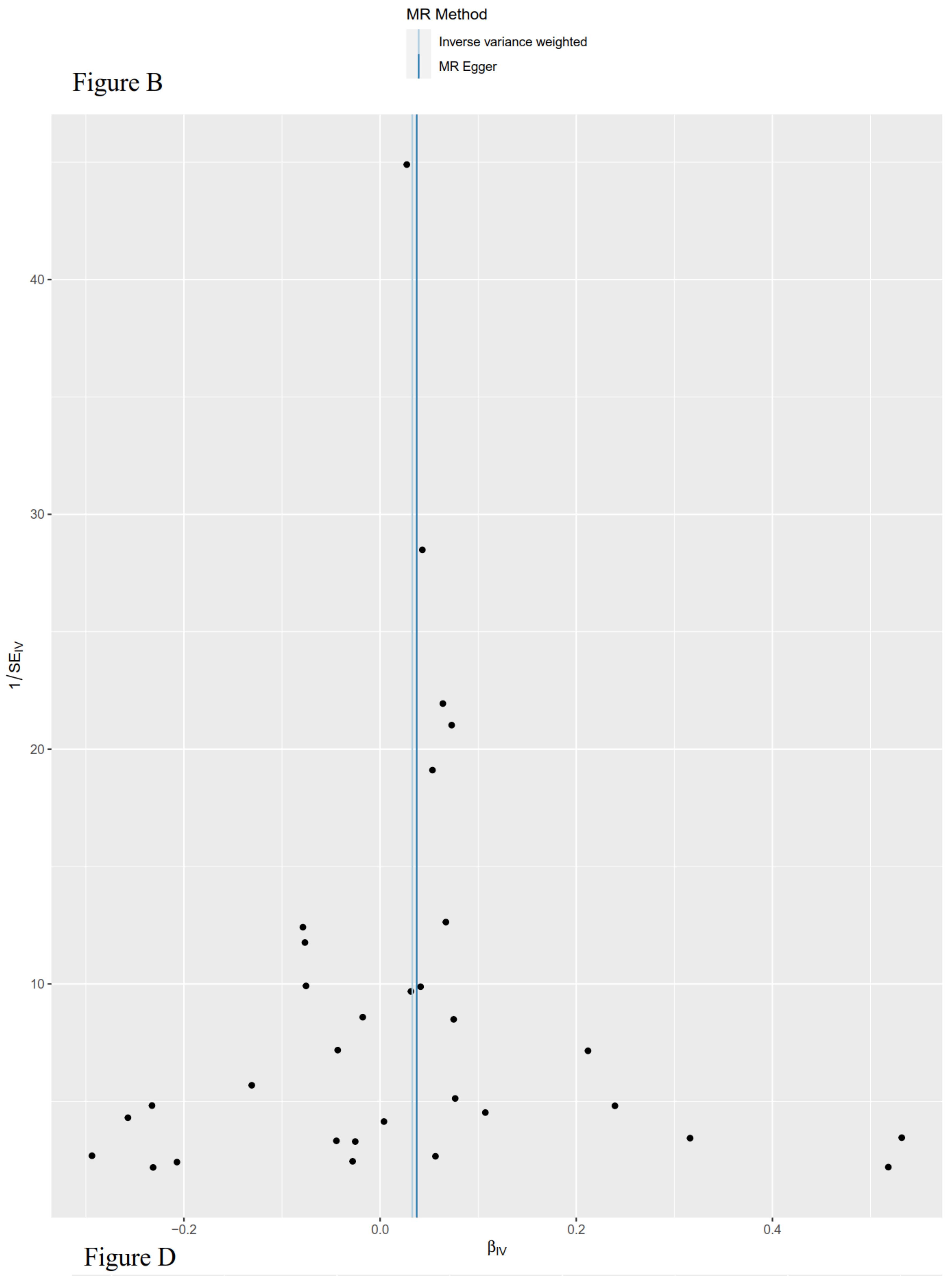

Figure C

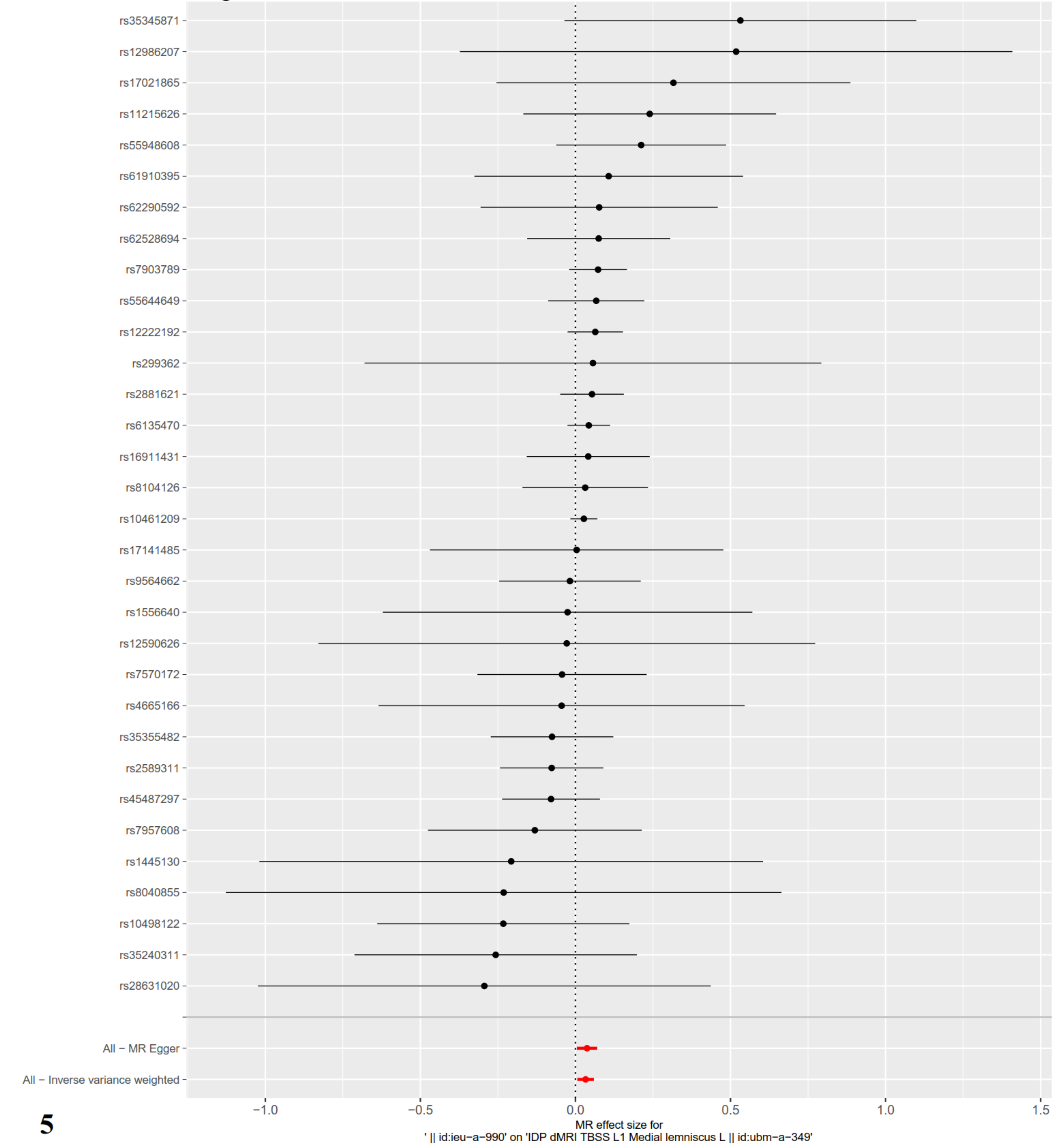

Figure D

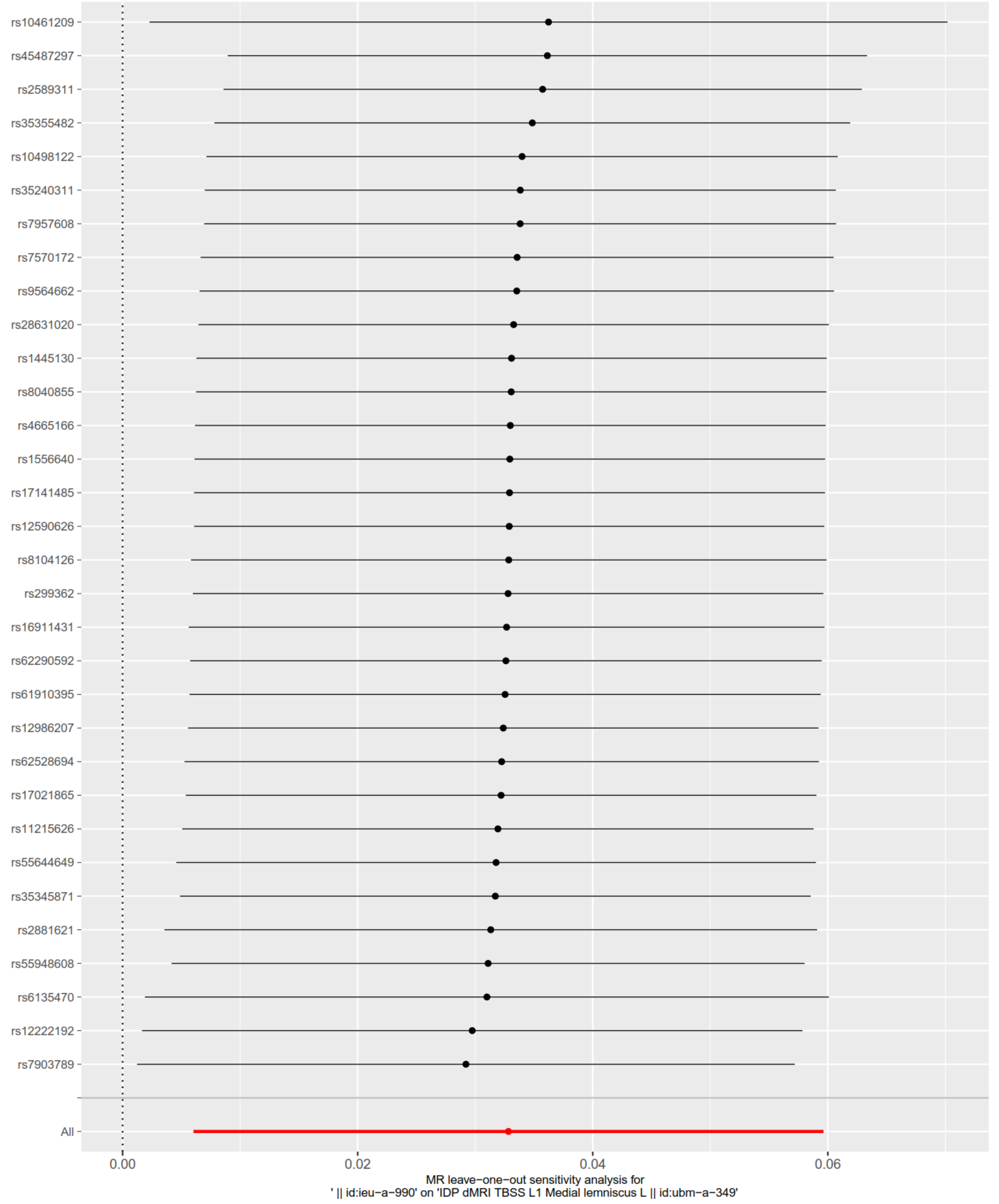

5

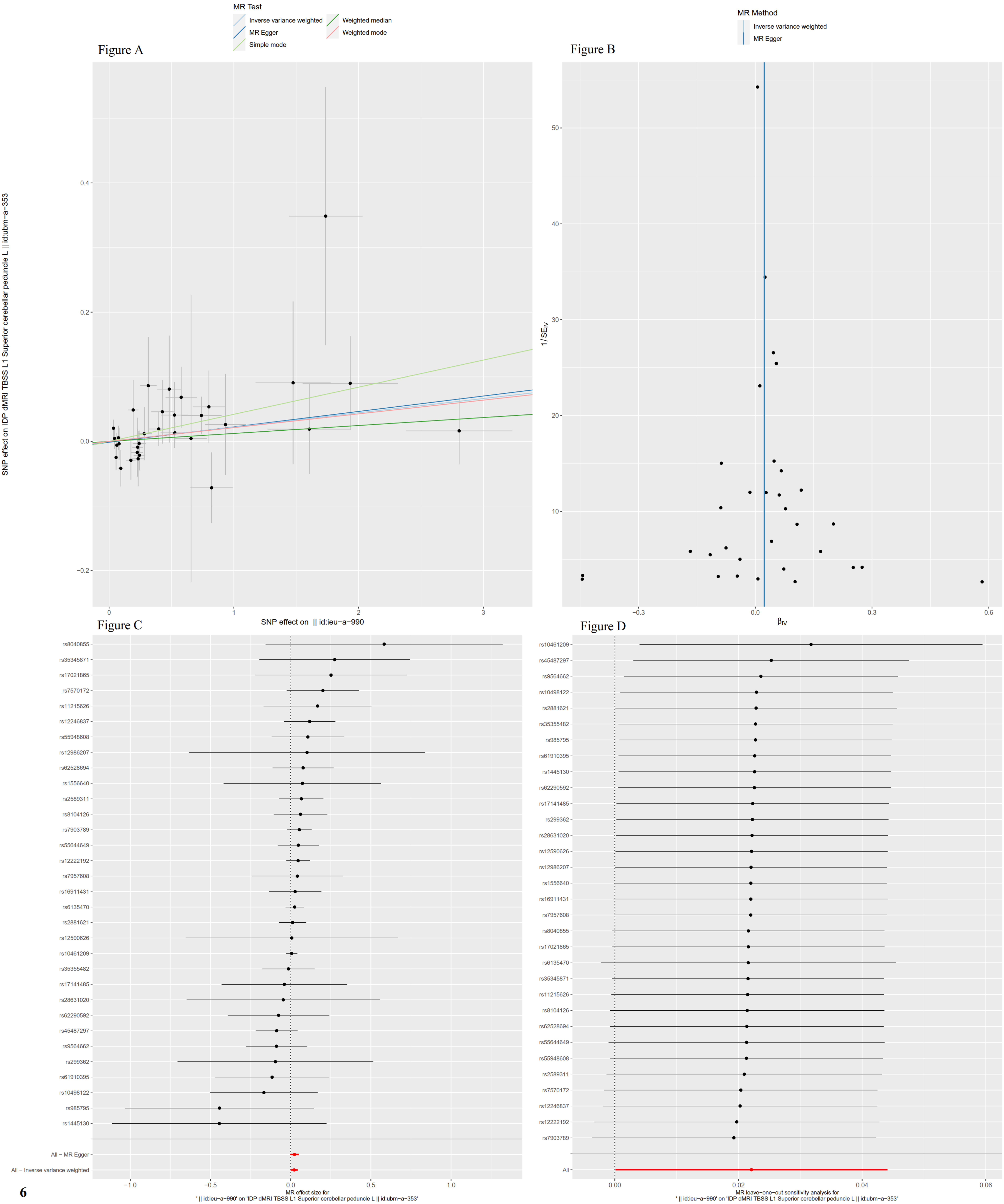
Figure A
MR Test
Inverse variance weighted
MR Egger
Simple mode
Weighted median
Weighted mode
SNP effect on IDP dMRI TBSS L1 Superior cerebellar peduncle L || id:ubm−a−353
SNP effect on || id:ieu−a−990
Figure B
MR Method
Inverse variance weighted
MR Egger
1/SE_IV
β_IV
Figure C
rs8040855
rs35345871
rs17021865
rs7570172
rs11215626
rs12246837
rs55948608
rs12986207
rs62528694
rs1556640
rs2589311
rs8104126
rs7903789
rs55644649
rs12222192
rs7957608
rs16911431
rs6135470
rs2881621
rs12590626
rs10461209
rs35355482
rs17141485
rs28631020
rs62290592
rs45487297
rs9564662
rs299362
rs61910395
rs10498122
rs985795
rs1445130
All − MR Egger
All − Inverse variance weighted
MR effect size for
' || id:ieu−a−990' on 'IDP dMRI TBSS L1 Superior cerebellar peduncle L || id:ubm−a−353'
Figure D
rs10461209
rs45487297
rs9564662
rs10498122
rs2881621
rs35355482
rs985795
rs61910395
rs1445130
rs62290592
rs17141485
rs299362
rs28631020
rs12590626
rs12986207
rs1556640
rs16911431
rs7957608
rs8040855
rs17021865
rs6135470
rs35345871
rs11215626
rs8104126
rs62528694
rs55644649
rs55948608
rs2589311
rs7570172
rs12246837
rs12222192
rs7903789
All
MR leave−one−out sensitivity analysis for
' || id:ieu−a−990' on 'IDP dMRI TBSS L1 Superior cerebellar peduncle L || id:ubm−a−353'

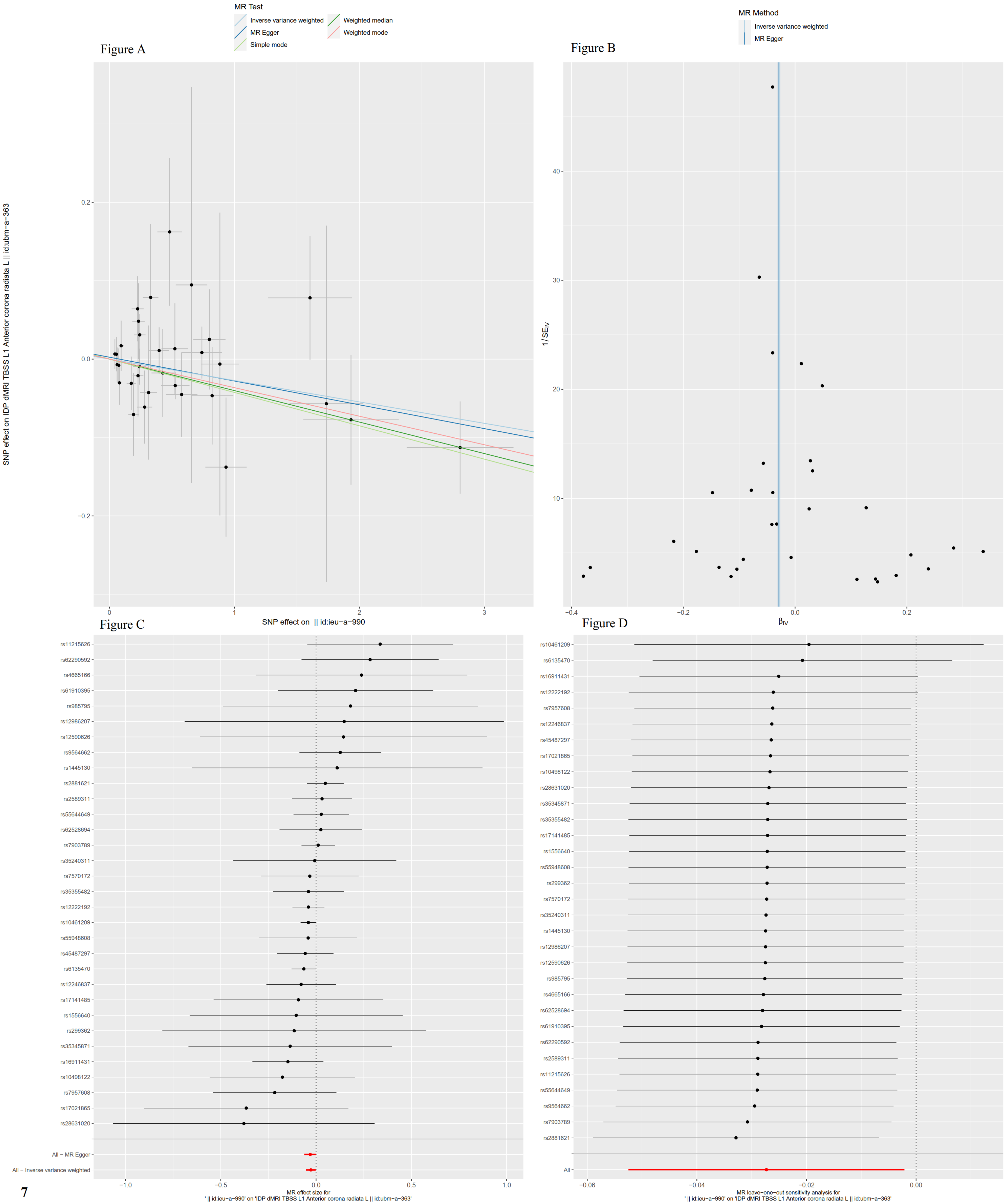
Figure A
MR Test
Inverse variance weighted
MR Egger
Simple mode
Weighted median
Weighted mode
SNP effect on IDP dMRI TBSS L1 Anterior corona radiata L || id:ubm-a-363
SNP effect on || id:ieu-a-990
Figure B
MR Method
Inverse variance weighted
MR Egger
$1/SE_{IV}$
β_{IV}
Figure C
rs11215626
rs62290592
rs4665166
rs61910395
rs985795
rs12986207
rs12590626
rs9564662
rs1445130
rs2881621
rs2589311
rs55644649
rs62528694
rs7903789
rs35240311
rs7570172
rs35355482
rs12222192
rs10461209
rs55948608
rs45487297
rs6135470
rs12246837
rs17141485
rs1556640
rs299362
rs35345871
rs16911431
rs10498122
rs7957608
rs17021865
rs28631020
All − MR Egger
All − Inverse variance weighted
MR effect size for
' || id:ieu-a-990' on 'IDP dMRI TBSS L1 Anterior corona radiata L || id:ubm-a-363'
Figure D
rs10461209
rs6135470
rs16911431
rs12222192
rs7957608
rs12246837
rs45487297
rs17021865
rs10498122
rs28631020
rs35345871
rs35355482
rs17141485
rs1556640
rs55948608
rs299362
rs7570172
rs35240311
rs1445130
rs12986207
rs12590626
rs985795
rs4665166
rs62528694
rs61910395
rs62290592
rs2589311
rs11215626
rs55644649
rs9564662
rs7903789
rs2881621
All
MR leave−one−out sensitivity analysis for
' || id:ieu-a-990' on 'IDP dMRI TBSS L1 Anterior corona radiata L || id:ubm-a-363'

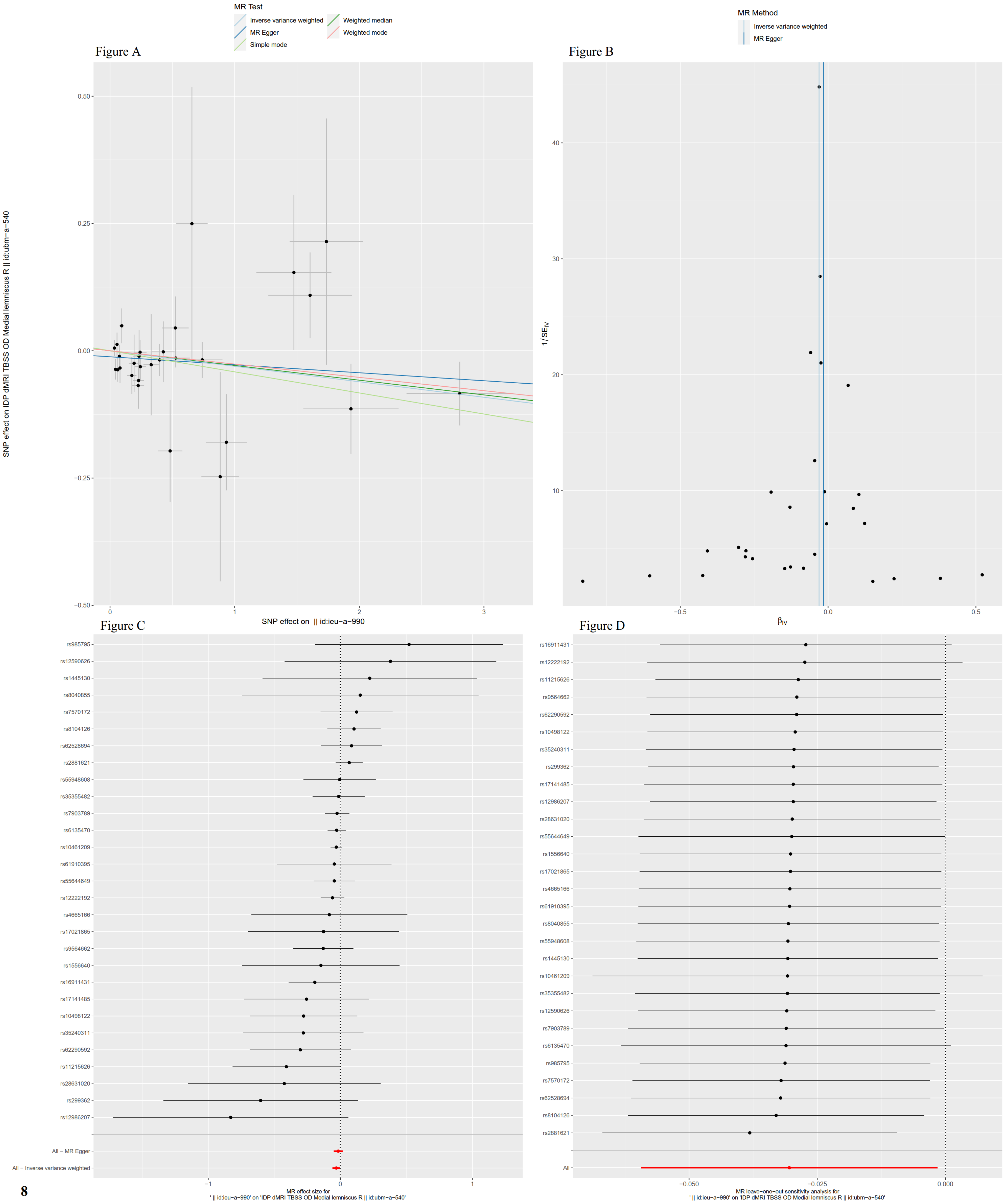

MR Test
Inverse variance weighted
MR Egger
Simple mode
Weighted median
Weighted mode
Figure A
SNP effect on IDP dMRI TBSS OD Medial lemniscus R || id:ubm-a-540
SNP effect on || id:ieu-a-990
MR Method
Inverse variance weighted
MR Egger
Figure B
1/SE_IV
β_IV
Figure C
rs985795
rs12590626
rs1445130
rs8040855
rs7570172
rs8104126
rs62528694
rs2881621
rs55948608
rs35355482
rs7903789
rs6135470
rs10461209
rs61910395
rs55644649
rs12222192
rs4665166
rs17021865
rs9564662
rs1556640
rs16911431
rs17141485
rs10498122
rs35240311
rs62290592
rs11215626
rs28631020
rs299362
rs12986207
All − MR Egger
All − Inverse variance weighted
MR effect size for
' || id:ieu-a-990' on 'IDP dMRI TBSS OD Medial lemniscus R || id:ubm-a-540'
Figure D
rs16911431
rs12222192
rs11215626
rs9564662
rs62290592
rs10498122
rs35240311
rs299362
rs17141485
rs12986207
rs28631020
rs55644649
rs1556640
rs17021865
rs4665166
rs61910395
rs8040855
rs55948608
rs1445130
rs10461209
rs35355482
rs12590626
rs7903789
rs6135470
rs985795
rs7570172
rs62528694
rs8104126
rs2881621
All
MR leave−one−out sensitivity analysis for
' || id:ieu-a-990' on 'IDP dMRI TBSS OD Medial lemniscus R || id:ubm-a-540'

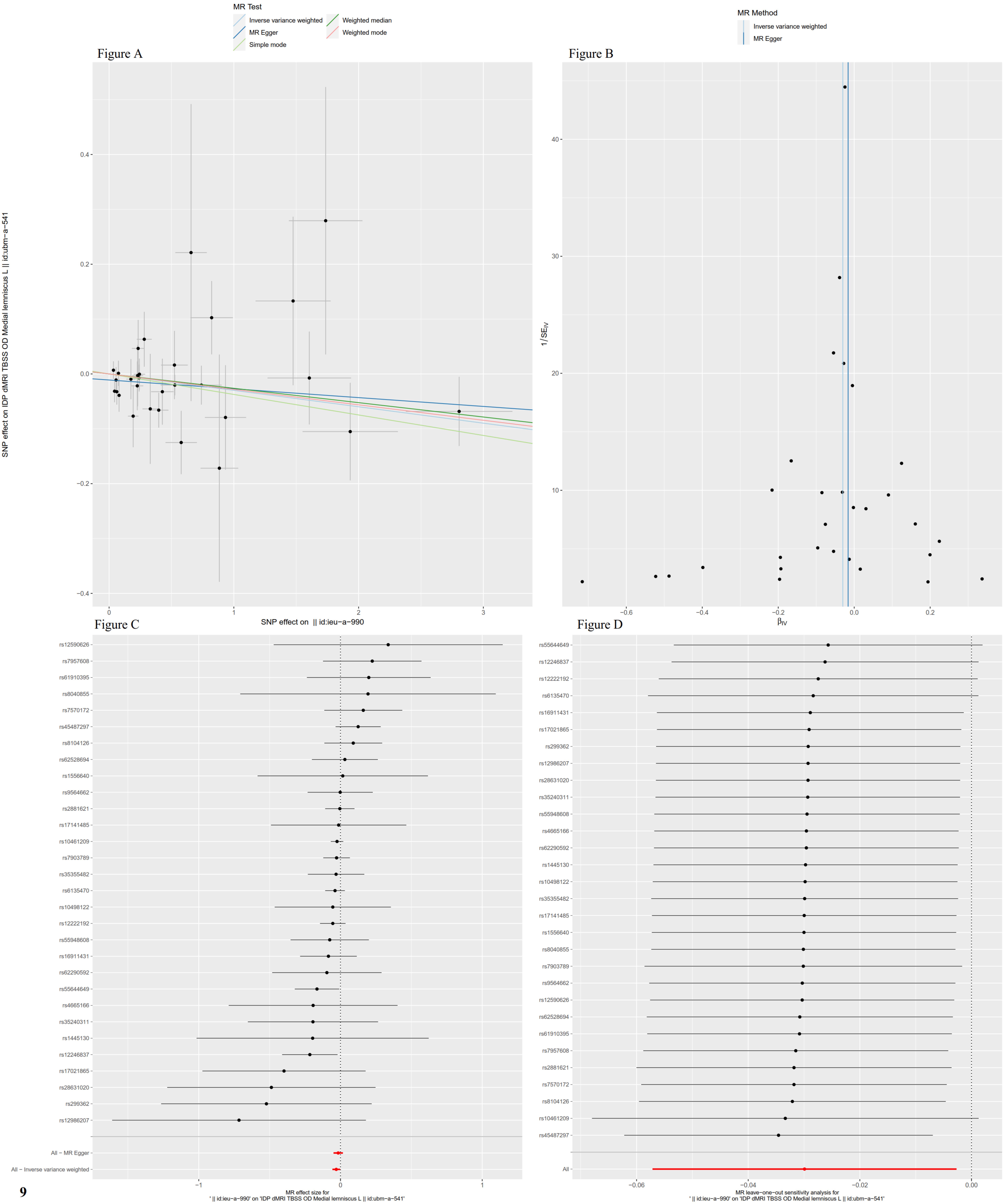
MR Test
Inverse variance weighted
MR Egger
Simple mode
Weighted median
Weighted mode
Figure A
SNP effect on IDP dMRI TBSS OD Medial lemniscus L || id:ubm−a−541
SNP effect on || id:ieu−a−990
MR Method
Inverse variance weighted
MR Egger
Figure B
1/SE_IV
β_IV
Figure C
rs12590626
rs7957608
rs61910395
rs8040855
rs7570172
rs45487297
rs8104126
rs62528694
rs1556640
rs9564662
rs2881621
rs17141485
rs10461209
rs7903789
rs35355482
rs6135470
rs10498122
rs12222192
rs55948608
rs16911431
rs62290592
rs55644649
rs4665166
rs35240311
rs1445130
rs12246837
rs17021865
rs28631020
rs299362
rs12986207
All − MR Egger
All − Inverse variance weighted
MR effect size for
' || id:ieu−a−990' on 'IDP dMRI TBSS OD Medial lemniscus L || id:ubm−a−541'
Figure D
rs55644649
rs12246837
rs12222192
rs6135470
rs16911431
rs17021865
rs299362
rs12986207
rs28631020
rs35240311
rs55948608
rs4665166
rs62290592
rs1445130
rs10498122
rs35355482
rs17141485
rs1556640
rs8040855
rs7903789
rs9564662
rs12590626
rs62528694
rs61910395
rs7957608
rs2881621
rs7570172
rs8104126
rs10461209
rs45487297
All
MR leave−one−out sensitivity analysis for
' || id:ieu−a−990' on 'IDP dMRI TBSS OD Medial lemniscus L || id:ubm−a−541'
